# Supplementary material for: A therapeutic approach to pantothenate kinase associated neurodegeneration
Source: Nat Commun. 2018 Oct 23;9:4399. doi: 10.1038/s41467-018-06703-2 (PMC6199309; doi:10.1038/s41467-018-06703-2)
Supplement: Supplementary file 1 — Supplementary Information [file 41467_2018_6703_MOESM1_ESM.pdf]

## Supplementary Information

# A Therapeutic Approach to Pantothenate Kinase Associated Neurodegeneration

Lalit Kumar Sharma[1,2], Chitra Subramanian[1], Mi-Kyung Yun[3], Matthew W. Frank[1], Stephen White[3], Charles O. Rock[1*], Richard E. Lee[2], Suzanne Jackowski[1*]
[1]Department of Infectious Diseases,
[2]Department of Chemical Biology and Therapeutics, and
[3]Department of Structural Biology, St. Jude Children's Research Hospital, Memphis, TN, USA

**Supplementary Table 1. Physicochemical properties and ADME parameters of PZ-2789, PZ-2891 and PZ-3067.**

| Property | PZ-2789 | PZ-2891 | PZ-3067 |
|---|---|---|---|
| $IC_{50(PANK3)}$ (nM) | 844 | 1.3 | >100,000 |
| Molecular Weight | 363.46 | 349.43 | 349.44 |
| ClogP* | 3.3 | 2.3 | 2.7 |
| LipE ($pIC_{50}$ – ClogP) | 2.7 | 6.6 | – |
| Solubility at pH 7.4 (μM) | 1.4 | 23 | 18 |
| Microsomal Stability $T_{1/2}$ (h) (m/r/h)** | 0.26/0.51/0.13 | 0.14/0.79/0.12 | 0.51/0.22/0.15 |
| % Protein Binding (m/r/h)** | 99/98/99 | 92/91/93 | 96/90/96 |
| Caco2 Permeability Efflux Ratio (B2A/A2B) | 0.5 | 0.5 | 0.5 |
| Plasma Stability $T_{1/2}$ (h) (m/r/h)** | >50/>50/>50 | >50/>50/>50 | >50/27/>50 |
| PAMPA; Avg Pe ($10^{-6}$ cm/s) | 1232 | 413 | 204 |
| PAMPA-BBB***; Avg Pe ($10^{-6}$cm/s) | 303 | 52 | 0.8 |

* Calculated using ChemBioDraw Ultra
**m/r/h = mouse/rat/human
***BBB = Blood Brain Barrier

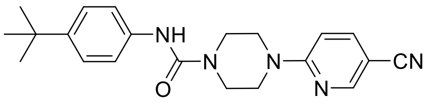


**PZ-2789**
**(hit from the screen)**

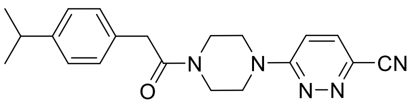


**PZ-2891**
**(optimized compound)**

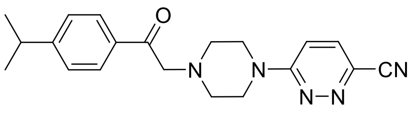


**PZ-3067**
**(negative control)**

**Supplementary Table 2. PZ-2891 pharmacokinetics.** Mean pharmacokinetic parameters of PZ-2891 in plasma following single intravenous (Dose = 2 mg/kg) or oral (Dose = 10 mg/kg) administration to male BALB/c mice[a].

| Route[b] | Dose (mg/kg) | $T_{max}$ (hr) | $C_0$/ $C_{max}$[c] (ng/mL) | $AUC_{last}$ (hr*ng/mL) | $AUC_{inf}$ (hr*ng/mL) | $T_{1/2}$ (hr) | CL (mL/min/kg) | Vss (L/kg) | %F[d] |
|---|---|---|---|---|---|---|---|---|---|
| i.v. | 2 | – | 2808.32 | 608.58 | 611.48 | 0.28 | 54.51 | 0.94 | **46** |
| p.o. | 10 | 0.25 | 1886.47 | 1397.83 | 1424.03 | – | – | – | |

[a]Pharmacokinetics measurements performed by Sai Life Sciences Limited.
[b]30% captisol was used to formulate PZ-2891.
[c]Back extrapolated concentration for i.v. group.
[d]Bioavailability was calculated based on $AUC_{last}$.

**Supplementary Table 3**. **Crystallography data collection and refinement statistics.**

| PANK3•AMPPNP•$Mg^{2+}$•PZ-2891 Complex | |
|---|---|
| **Data collection** | |
| Space group | $P3_121$ |
| Cell dimensions | |
| *a*, *b*, *c* (Å) | 98.9, 98.9, 69.1 |
| α, β, γ (°) | 90.0, 90.0, 120.0 |
| Resolution (Å) | 50.0-1.6 (1.64-1.6)[a] |
| $R_{sym}$ or $R_{merge}$ | 0.061 (0.728) |
| $CC_{1/2}$ | 0.999 (0.712) |
| $I / \sigma I$ | 34.6 (1.7) |
| Completeness (%) | 99.7 (98.7) |
| Redundancy | 6.8 (4.4) |
| | |
| **Refinement** | |
| Resolution (Å) | 32.4-1.6 |
| No. reflections | 51,447 |
| $R_{work}$ / $R_{free}$ | 0.178 / 0.195 |
| No. atoms | |
| Protein | 2,720 |
| Ligand/ion | 65/1 |
| Water | 148 |
| *B*-factors | |
| Protein | 32.6 |
| Ligand/ion | 25.7/18.4 |
| Water | 40.6 |
| R.m.s. deviations | |
| Bond lengths (Å) | 0.007 |
| Bond angles (°) | 0.977 |

[a]Values in parentheses are for highest-resolution shell. Data were collected on a single crystal.

**Supplementary Table 4. Genotyping primers to identify *SynCre-Pank1,Pank2* mice.** The primer sets were used to detect the presence or absence of the *SynCre* transgene, and to detect the presence of the floxed and knockout alleles of the *Pank1* and *Pank2* genes as described in Supplementary Fig. 15.

| | | | |
|---|---|---|---|
| *Pank1* | *Pank1* F1 | GGATAGGATGGCTACTAGCTGC | 94°C, 5 min + 40 cycles (94°C, 30sec + 58°C, 30sec + 72°C, 30sec) + 72°C, 5 min + 4°C, ∞<br>*Pank1* F1 + *Pank1* R1 + *Pank1* R2<br>WT =274bp, Floxed =338bp, KO=218bp |
| | *Pank1* R1 | TTACTAGCTAAGTGGCCCAGG | |
| | *Pank1* R2 | GCCTAATTTCGTTCACAGTGG | |
| *Pank2* | *Pank2* F1 | CTGGAAATCTCGTGTAGTTGAGTC | 94°C, 5 min + 40 cycles (94°C, 30sec + 58°C, 30sec + 72°C, 1min) + 72°C, 5 min + 4°C, ∞<br>*Pank2* F1 + *PanK2* R1 + *PanK2* R2<br>WT =262bp, Floxed=332bp, KO=176bp |
| | *Pank2* R1 | TCAAGCAGTATCAAAGACACCAC | |
| | *Pank2* R2 | CACTGGAGCAGTAACTGAGAGC | |
| *Cre* | 15563 (*Cre*) | CTCAGCGCTGCCTCAGTCT | 94°C, 5 min + 45 cycles (94°C, 30sec + 60°C, 30sec + 72°C, 45sec) + 72°C, 5 min + 4°C, ∞<br>*Cre*=300bp, Internal Control=200bp |
| | 15564 (*Cre*) | GCATCGACCGGTAATGCA | |
| | IMR0015 (internal control) | CAAATGTTGCTTGTCTGGTG | |
| | IMR0016 (internal control) | GTCAGTCGAGTGCACAGTTT | |

**Supplementary Table 5. RT-qPCR primers for human and murine PanKs.** These primers were used to measure the abundance of the three *Pank* mRNAs in C3A cells in Supplementary Fig 9 and mouse tissues as shown in Supplementary Fig. 16. The human and mouse *Gapdh* primers used as the calibrator were purchased from Applied Biosystems (catalog # 402869-human) (catalog #4308313-mouse).

| | Gene | Forward Primer (5′-3′) | Reverse Primer (5′-3′) |
|---|---|---|---|
| *Murine* | *Pank1* Exon 4 | TCAGGGCCTTCTTTACGTTG | TGGAGTACACTGCCAGGATG |
| | *Pank2* Exon 3 | CCCTGCTGATTCTGAAAAATG | GAGCCGATGTTCACCAGAAG |
| | *Pank3* Exon3 | AACCTCCACCTGCACAAACT | TGGGTAAGGATCATCCAGGT |
| *Human* | *PANK1* | TGAGCTTATTGTTGAAAGGGATTTT | GGTGCTCAGAAGGCAGAGAAA |
| | *PANK2* | AGAGCTGCTCTGTGTTCAGTTGA | CGCATAGTAGCCTGCCTTACATT |
| | *PANK3* | CACCATGCTCTTATCTCGCACTT | TTGAAGGCAGAGGGCTCAAC |

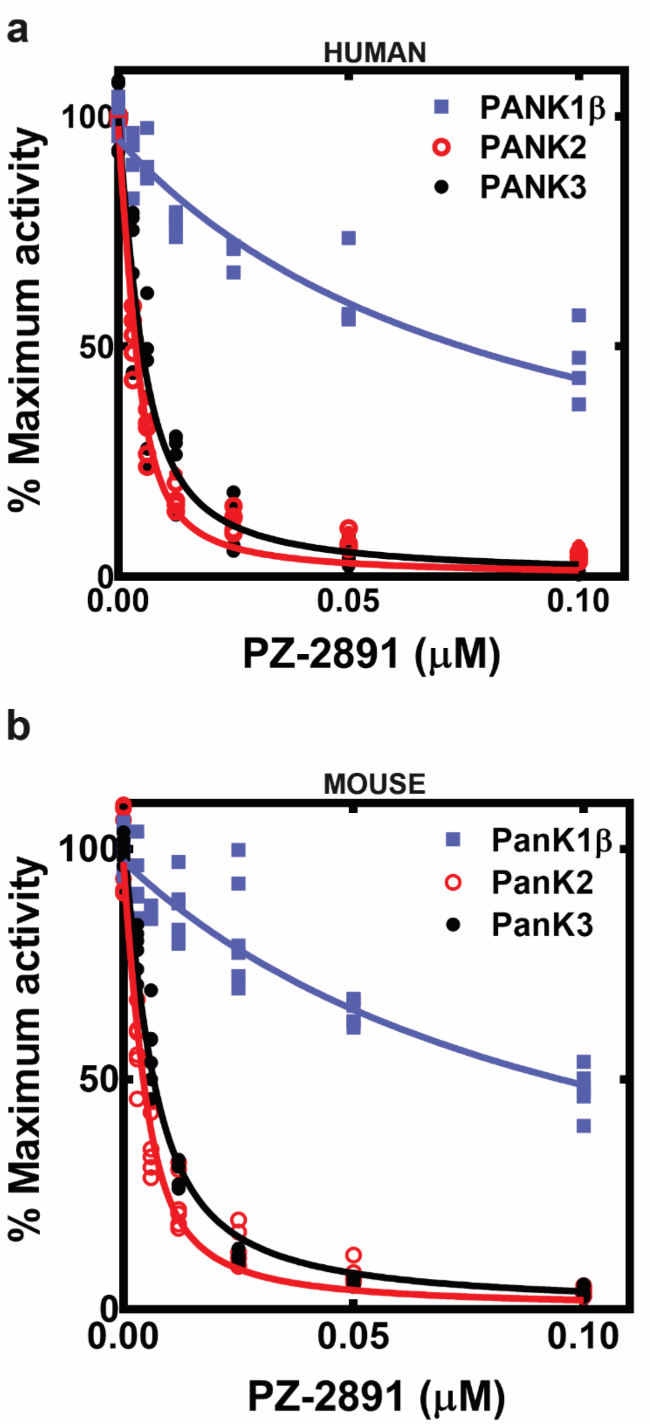


**Supplementary Fig. 1. Sensitivity of human and mouse pantothenate kinase isoforms to PZ-2891.** The $IC_{50}$ for PZ-2891 was determined for each of the purified His-tagged isoforms using the radiochemical kinase assay. The human PANK2 isoform was the mature, processed protein. The combined inhibition data were fitted using the Morrison equation. (**a**) Human pantothenate kinases. The calculated $IC_{50}$s in these experiments were: PANK1β, 40.2 ± 4.4 nM; PANK2, 0.7 ± 0.08 nM; and PANK3, 1.3 ± 0.2 nM. (**b**) Mouse pantothenate kinases. The calculated $IC_{50}$s in these experiments were: PanK1β, 48.7 ± 5.1 nM; PanK2, 1.0 ± 0.1 nM; and PanK3, 1.9 ± 0.2 nM.

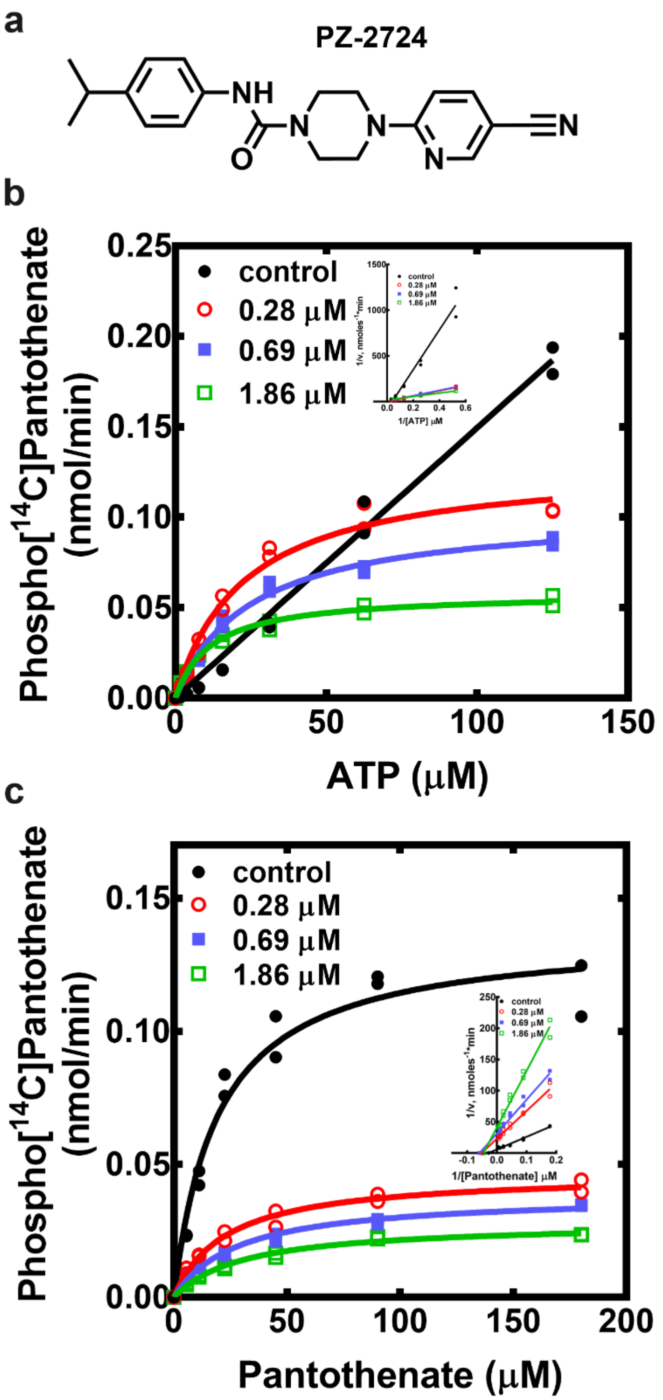


**Supplementary Fig. 2. Kinetic analysis of PANK3 inhibition by pantazines.** (**a**) Structure of PZ-2724 used in the kinetic analysis. Data were fit to the nonlinear Michaelis-Menten equation and the three concentrations of PZ-2724 and the double-reciprocal plots are shown in the insets. (**b**) Kinetics of pantazine inhibition with respect to ATP. Pantazine inhibition was uncompetitive with respect to ATP resulting in a lower Km for ATP and a lower Vmax. The graph focuses on the low concentrations of ATP to clearly illustrate the dual effect of pantazines on Vmax and Km. (**c**) Kinetics of inhibition with respect to pantothenate. Pantazine inhibition was noncompetitive with respect to pantothenate. The experiments shown are representative of two independent kinetic experiments performed in duplicate. Data were fit and kinetic parameters calculated using GraphPad software.

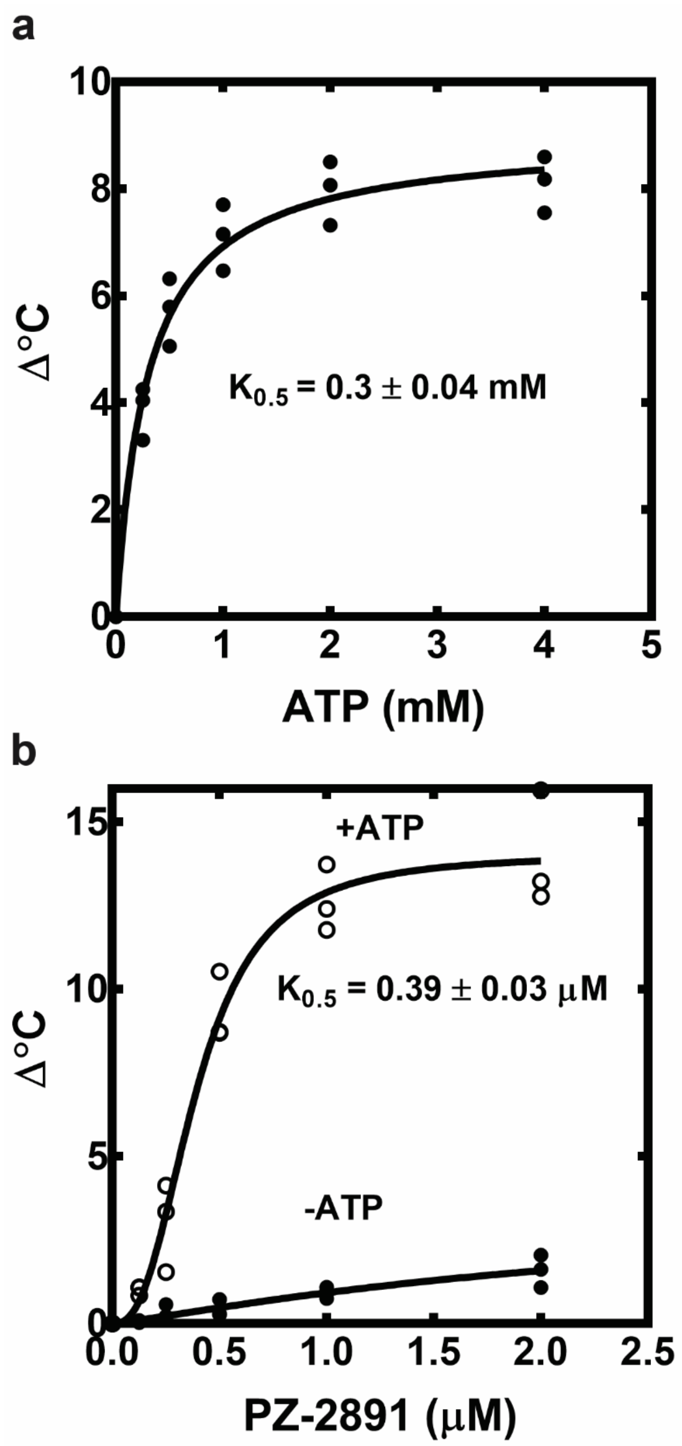


**Supplementary Fig. 3. Dose-response data for the thermal stabilization of PANK3 by ATP and PZ-2891.** The denaturation temperatures for PANK3 (2.5 μM) were determined at each indicated concentration of ligand as described under Methods. The concentration of ligand that stabilized 50% of the PANK3 ($K_{0.5}$) was calculated by fitting the combined data to the Michaelis-Menten equation. (**a**) ATP dependent stabilization of PANK3. (**b**) Stabilization of PANK3 by PZ-2891 in the presence and absence of 2.5 mM ATP. Data are from triplicate measurements. Without ATP, filled circles; with ATP + PZ-2891 open circles.

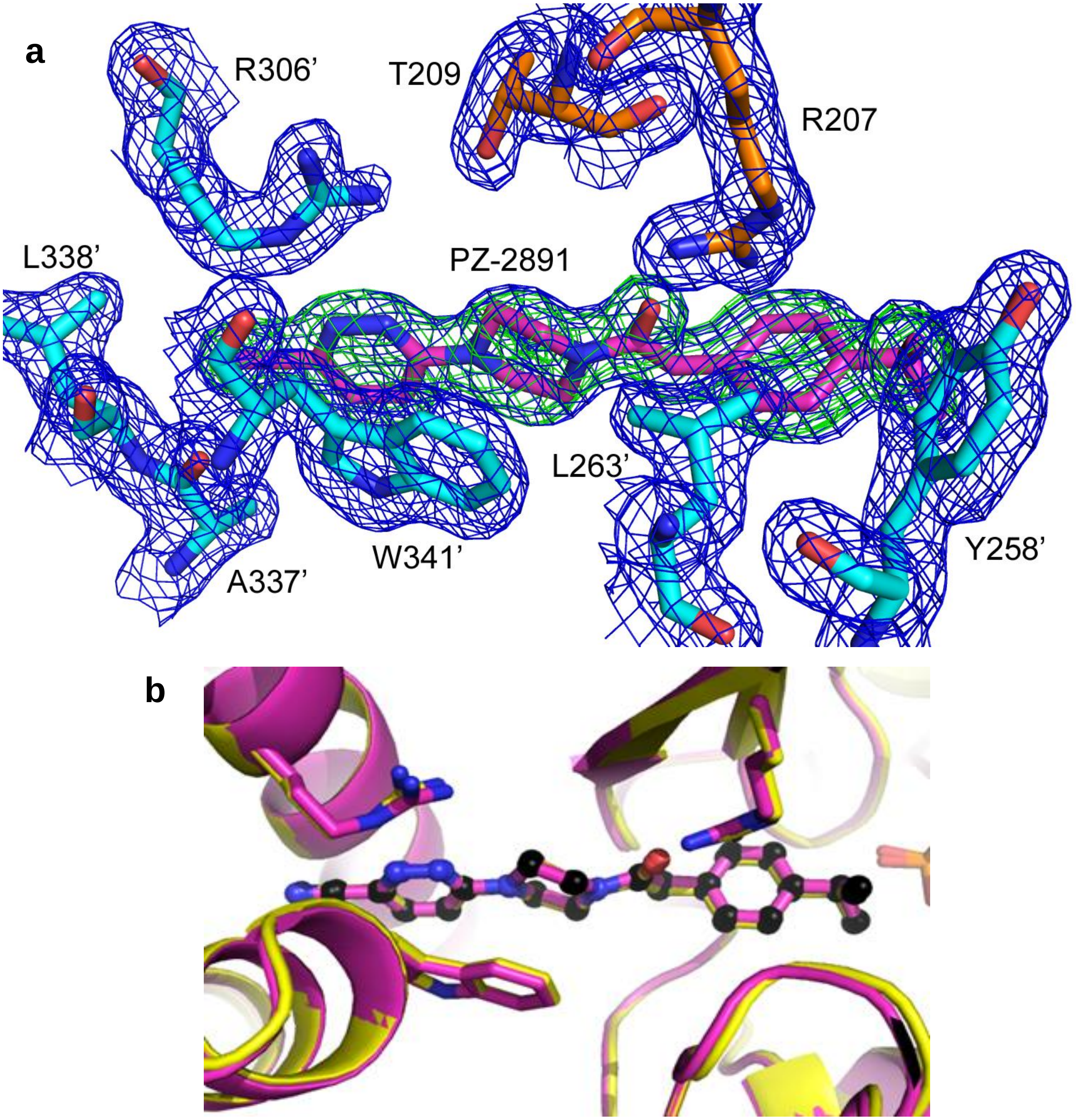


**Supplementary Fig. 4. Electron density maps for PZ-2891 binding site.** (**a**) The 2Fo-Fc electron density map (blue mesh) contoured at 1 $\sigma$ around PZ-2891 and the key residues that interact with PZ-2891. The Fo-Fc simulated annealing omit map (green mesh) was generated from refined structure in which PZ-2891 was omitted, and is contoured at 3 $\sigma$. (**b**) We also determined the PANK3•ATP•$Mg^{2+}$•PZ-2891 structure by co-crystallization at 2.5 Å resolution with final $R_{work}$ and $R_{free}$ values of 18.9% and 22.4%, respectively. The structures of the complex obtained by co-crystallization (magenta) and soaking (yellow) are overlaid, and are almost identical.

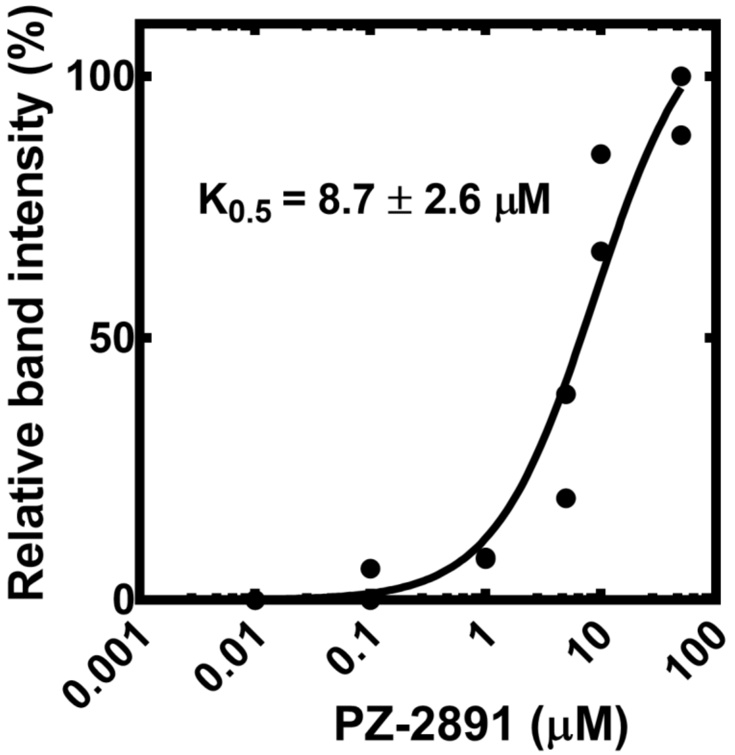


**Supplementary Fig. 5. PZ-2891 dose-response in the CETSA assay.** HEK293T cells overexpressing PANK3 were treated with the indicated concentrations of PZ-2891 and the amounts of stabilized PANK3 protein were determined using the CETSA assay at 62 °C as described under Methods. At this temperature, PZ-2891-bound PANK3 was stable in the CETSA assay, whereas the unbound PANK3 was not (Fig. 4b). The apparent $K_{0.5}$ for the stabilization of PANK3 by PZ-2891 was calculated using GraphPad software. The data are from two independent experiments.

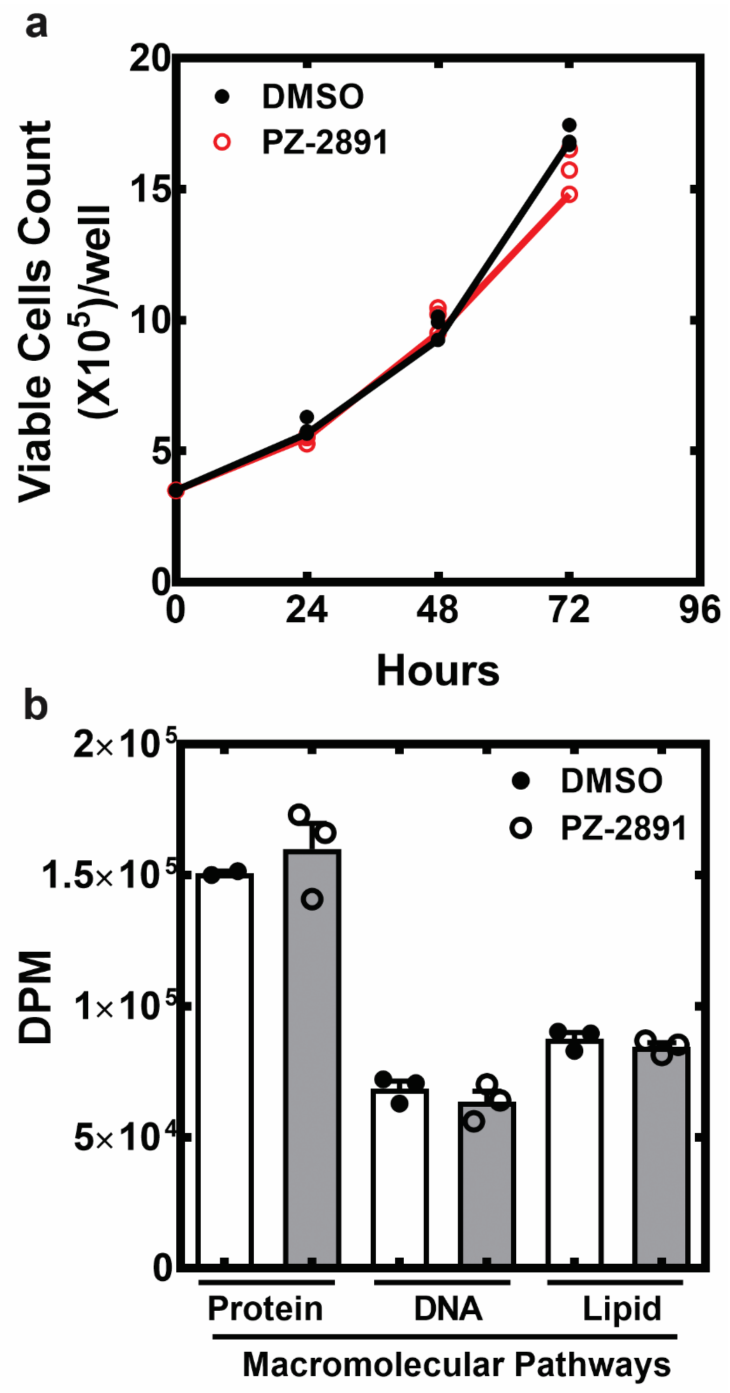


**Supplementary Fig. 6. Cell growth and macromolecular synthesis in PZ-2891-treated cells.** (**a**) A representative cell growth of C3A cells in the presence and absence of 10 μM PZ-2891. Black, control, red plus PZ-2891. (**b**) Pathway labeling of C3A cells treated with either DMSO or DMSO plus 10 μM PZ-2891. Protein synthesis was determined by measuring the incorporation of $^{3}$H-labeled amino acid mixture, DNA synthesis was determined by measuring the incorporation of [$^{3}$H]thymidine, and lipid synthesis was determined by labeling with [U-$^{14}$C]glucose and measuring the incorporation of label into the lipid fraction as described in Methods. Triplicate dishes of cells were used and the means ± SEM are plotted. There were no differences between control and PZ-2891 conditions. Open bars are control and gray bars are plus PZ-2891.

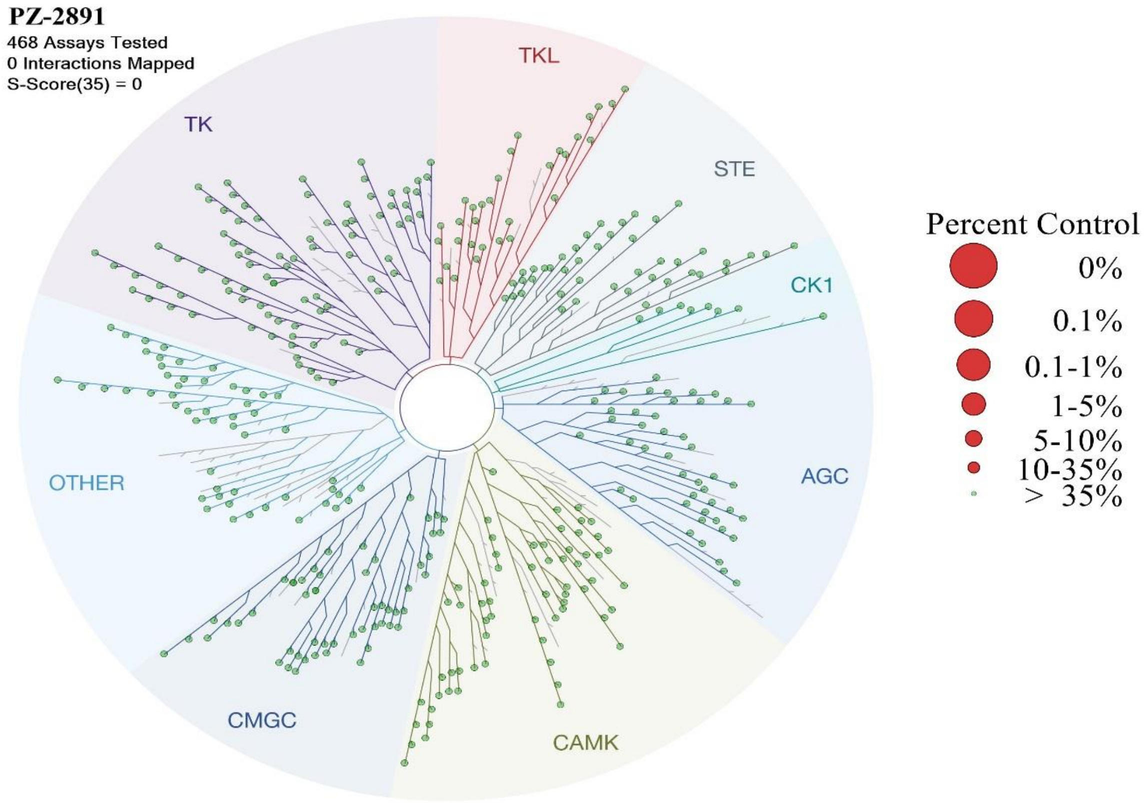


**Supplementary Fig. 7. Activity of PZ-2891 as an inhibitor of 468 kinases.** The KINOMEscan was performed by DiscoverX using 10 μM PZ-2891 against 468 human kinases. The Kinase panel covers AGC, CAMK, CMGC, CK1, STE, TK, TKL, lipid and atypical kinase families, plus important mutant forms. The treespot plot illustrates that PZ-2891 did not exhibit off-target inhibition of this kinase set. Data are provided as Supplementary Dataset 1.

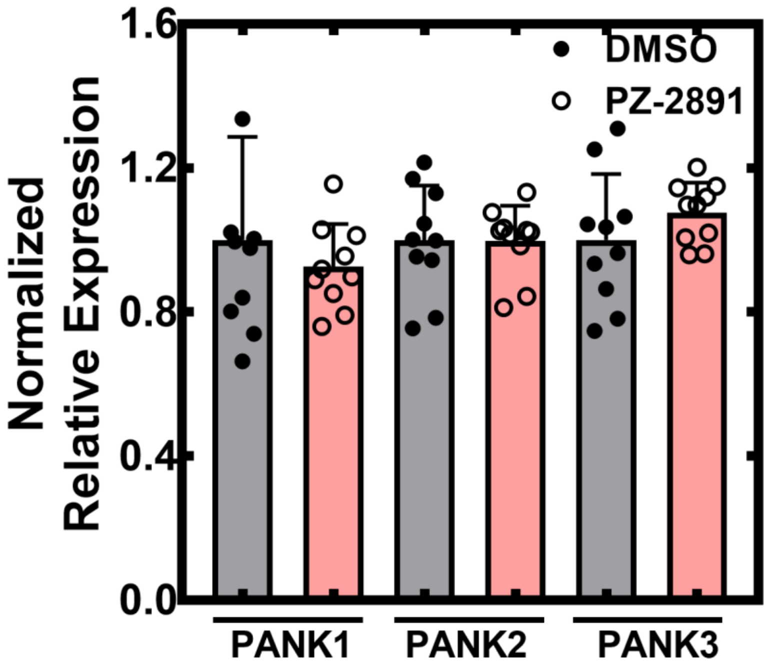


**Supplementary Figure 8. Pantothenate kinase transcript levels in control and PZ-2891-treated human C3A cells.** The levels of the three human PANK mRNAs were quantified by RT-qPCR using primers described previously (1). C3A cells were treated with either DMSO or DMSO containing 10 μM PZ-2891. RNAs from triplicate dishes were extracted, and mRNA levels quantitated by RT-qPCR in triplicated for each sample. Data are mean ± SEM. Primers are listed under the Cell Culture and Radiolabeling section in the Methods. Gray bars are control and pink bars are plus PZ-2891.

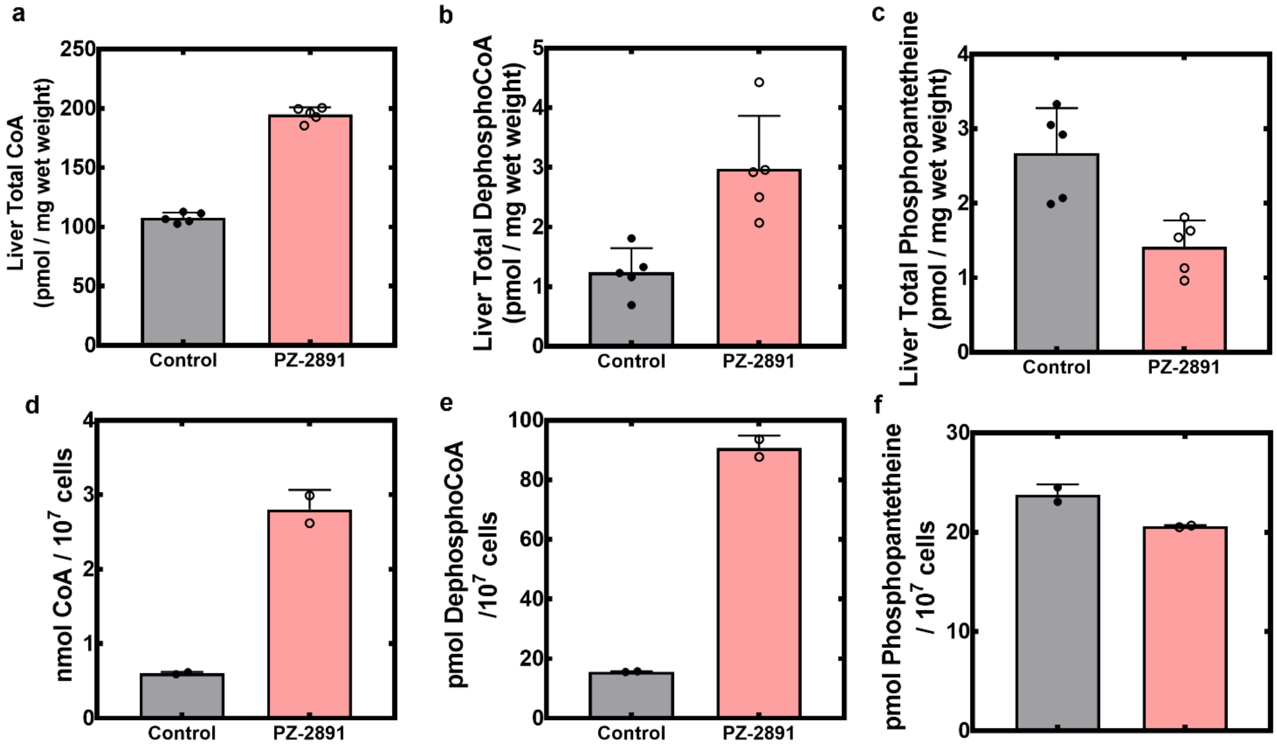


**Supplementary Fig. 9. Levels of CoA, dephospho-CoA and phosphopantetheine.** The levels of these three molecules were determined by HPLC of their fluorescent derivatives as described in Methods in triplicate samples of liver or C3A cells. Levels of (**a**) CoA, (**b**) dephosphoCoA and (**c**) phosphopantetheine in the livers of mice treated with 20 mg/kg PZ-2891 compared to control mice (5 mice per group). Levels of (**d**) CoA, (**e**) dephosphoCoA and (**f**) phosphopantetheine in cultured C3A treated with 10 μM PZ-2891 for 24 h compared to untreated controls. The experiment was replicated in duplicate. Means ± SEM are plotted. Gray bars as control and pink bars are with PZ-2891.

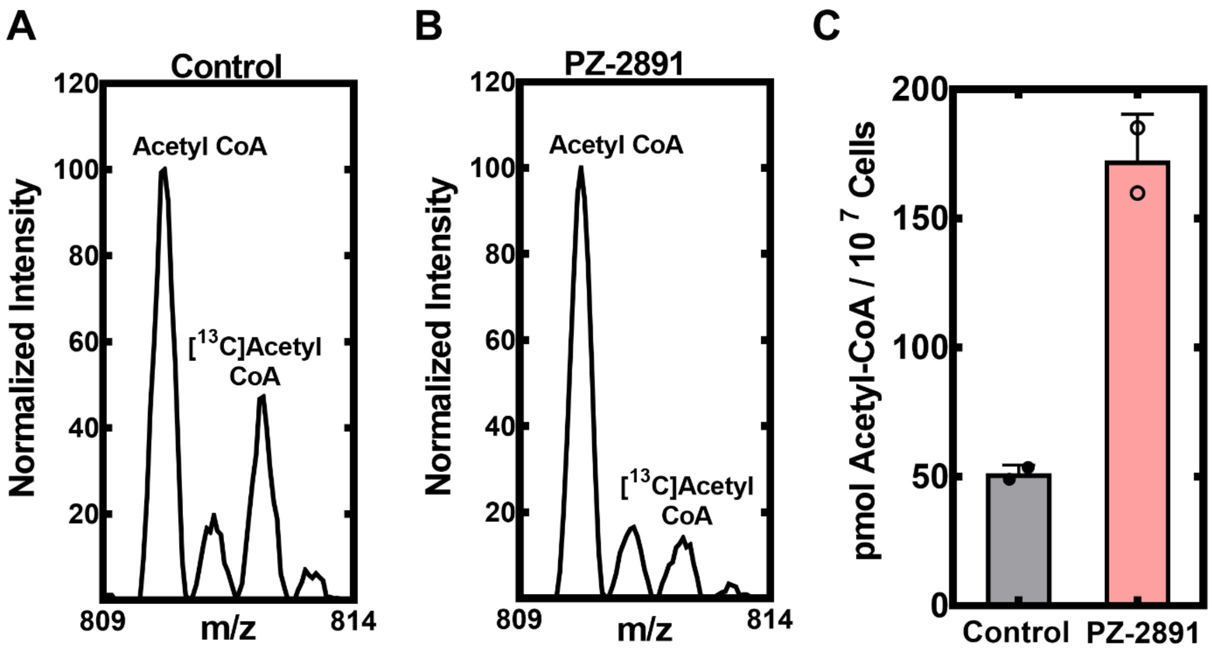


**Supplementary Fig. 10. PZ-2891 elevated acetyl-CoA in C3A cells.** C3A cells were treated for 24 h with 10 μM PZ-2891, and the cellular metabolites extracted with the addition of a [$^{13}$C]acetyl-CoA internal standard to quantify the acetyl-CoA signal. (**a**) A representative mass spectrum showing the acetyl-CoA levels in control C3A cells. (**b**) A representative mass spectrum showing acetyl-CoA levels in PZ-2891-treated C3A cells. (**c**) Quantification of acetyl-CoA levels in control and PZ-2891-treated C3A cells. Data were from duplicate measurements. Gray bars are control and pink bars are with PZ-2891.

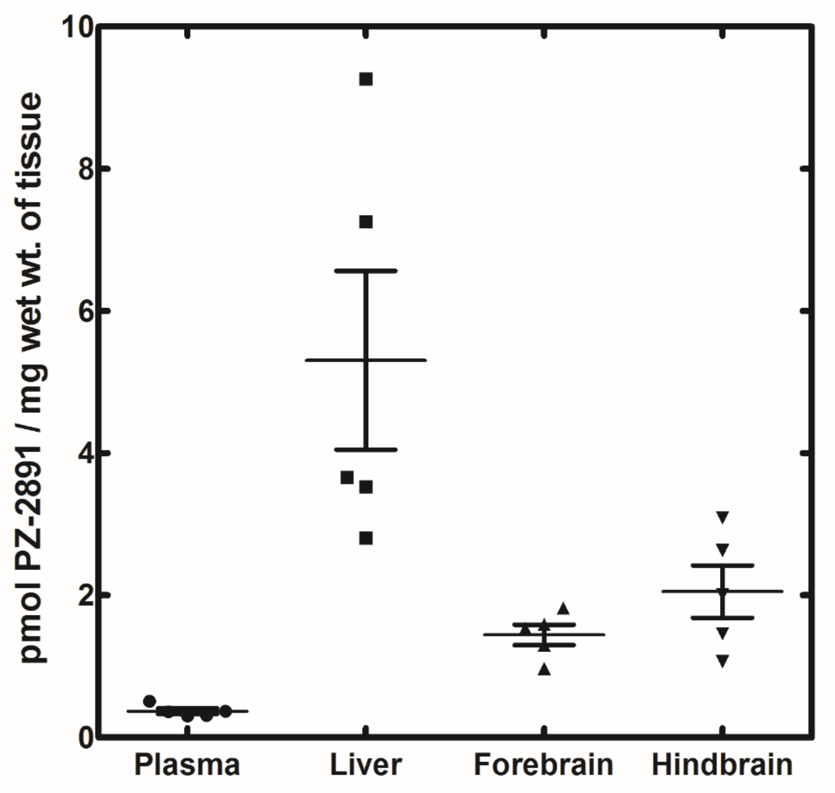


**Supplementary Fig. 11. PZ-2891 levels in plasma, liver and brain tissue.** Groups of 5 mice were maintained for 4 weeks on a diet containing 225 ppm PZ-2891 and 1000 ppm pantothenate. The mouse plasma, liver, forebrain and hindbrain were harvested, extracted and the levels of plasma and tissue PZ-2891 were determined by LC/MS/MS. Mean ± SEM is plotted.

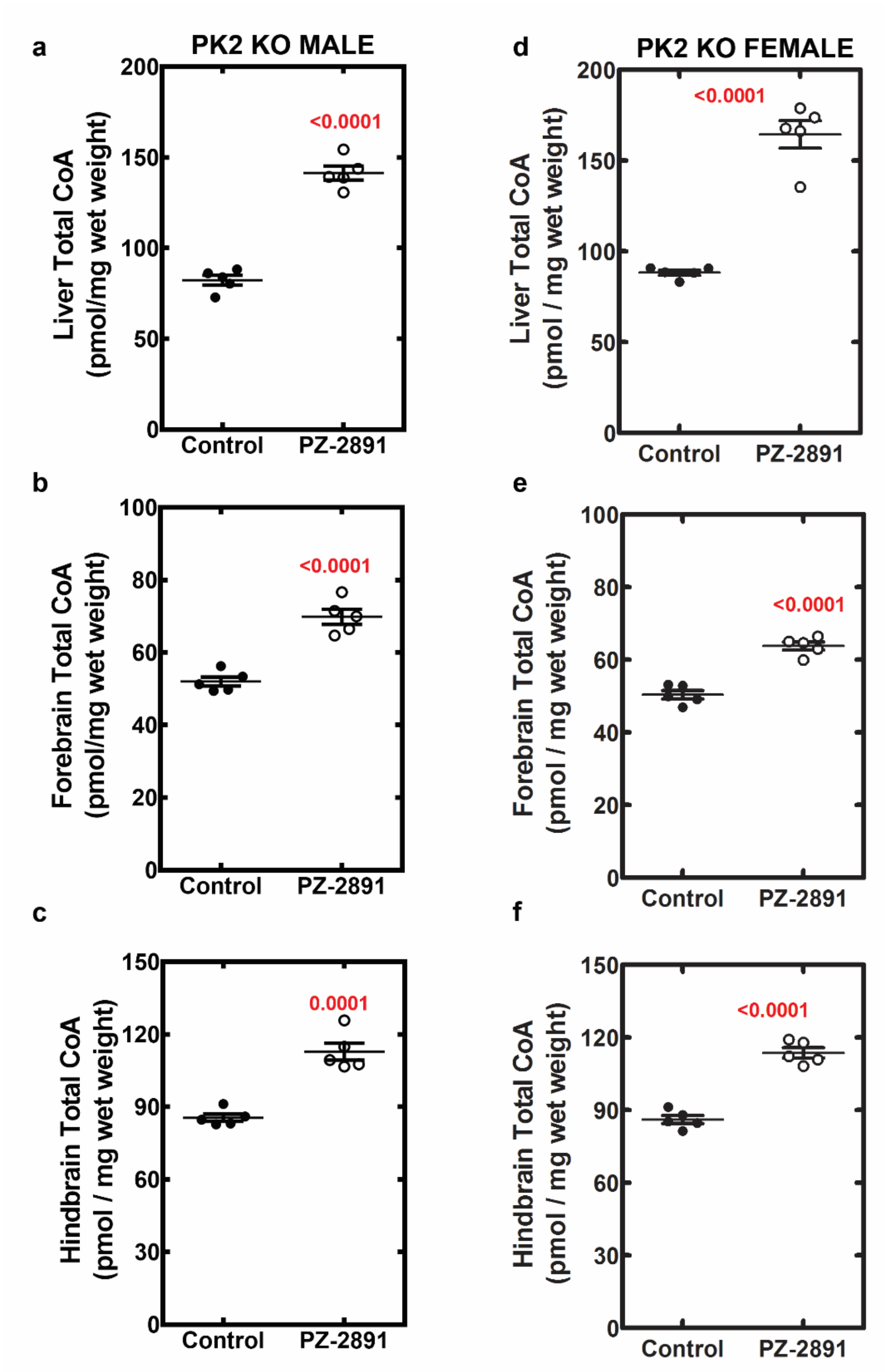


**Supplementary Fig. 12. PZ-2891 elevated CoA content in the tissues of *Pank2*$^{-/-}$ mice.** PZ-2891 elevation of CoA in liver (**a**), forebrain (**b**) and hindbrain (**c**) of male *Pank2*$^{-/-}$ mice (n=3). PZ-2891 elevation of CoA in liver (**d**), forebrain (**e**) and hindbrain (**f**) of female *Pank2*$^{-/-}$ mice (n=5). Groups of mice were maintained on a pantothenate (1000 ppm)-supplemented diet with or without PZ-2891 (225 ppm) for two weeks prior to analysis. Statistics were calculated using the Student's t-test. Mean ± SEM is plotted. Control, filled circles; with PZ-2891 open circles.

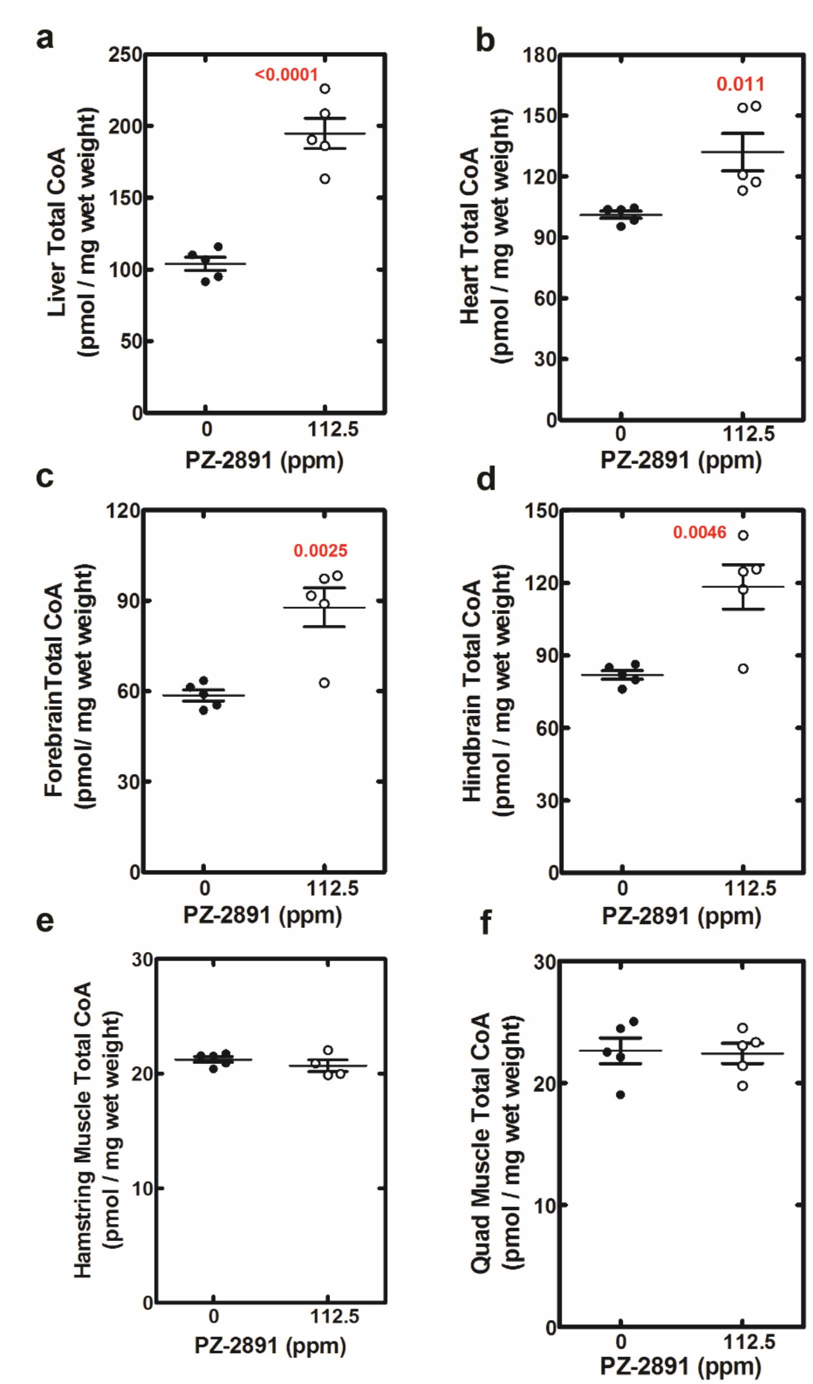


**Supplementary Fig. 13. Effect of PZ-2891 on tissue CoA.** Groups of 5 mice were maintained for 1 week on a diet containing 1000 ppm pantothenate with or without 112 ppm PZ-2891. The mouse tissues were harvested and the CoA levels were determined in 8 tissues. (**a**) Liver; (**b**) Forebrain; (**c**) Hindbrain; (**d**) Heart; (**e**) Hamstring muscle; (**f**) Quadriceps muscle. Statistical analysis of the data was performed using Student's *t* test and *p* values are indicated in red. Means ± SEM are plotted. Control, filled circles; with PZ-2891 open circles.

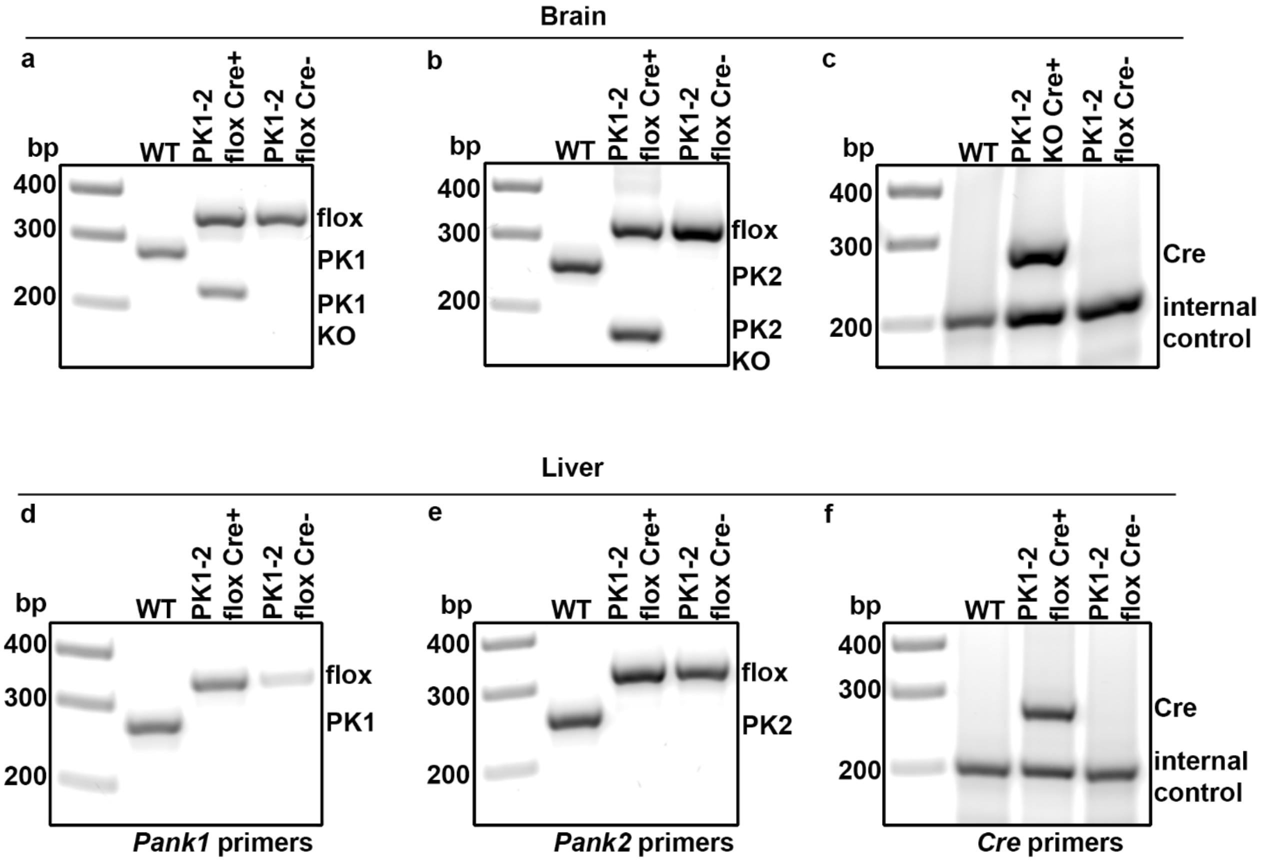


**Supplementary Figure 14. PCR genotyping of *SynCre Pank1,Pank2* neuronal knockout mice.** Genomic DNA was isolated from forebrains and livers of C57Bl6/J (**WT**), *SynCre+ Pank1$^{fl/fl}$,Pank2$^{fl/fl}$* (**PK1-2 flox Cre+**) and *SynCre– Pank1$^{fl/fl}$,Pank2$^{fl/fl}$* (**PK1-2 flox Cre**–) mice at postnatal Day 45. Primers specific for unfloxed wild-type and floxed *Pank1* and *Pank2* genes, and for the *SynCre* transgene, together with the sizes of the PCR products, are listed in Supplementary Table 4. Brain genotyping showed deletion of exon 4 in the *Pank1*-floxed gene (**a**), exon 3 in the *Pank2*-floxed gene (**b**) and the presence of the *Cre* recombinase transgene (**c**). Liver genotyping showed the lack of deletion and presence of an intact *Pank1*-floxed gene (**d**), intact *Pank2*-floxed gene (**e**) and the presence of the *SynCre* recombinase transgene (**f**). These data illustrate the specific inactivation of the *Pank1,2* genes in neurons of the central nervous system.

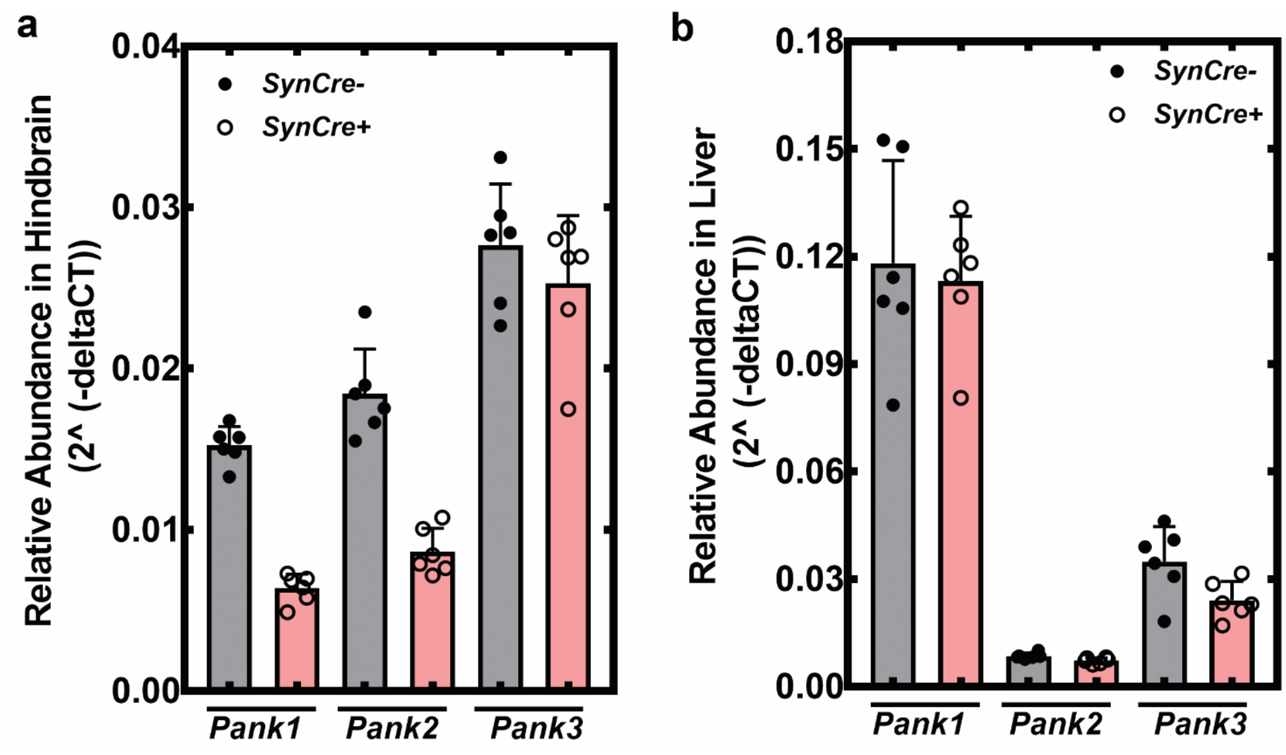


**Supplementary Figure 15. Transcript levels of pantothenate kinase isoforms in the liver and brain of *SynCre+ Pank1,Pank2* neuronal knockout mice.** The levels of the three *Pank* mRNAs were quantified by RT-qPCR using primers listed in Supplementary Table 5. RNAs were isolated from (**a**) hindbrain or (**b**) livers of 3 male and 3 female *SynCre– Pank1*$^{fl/fl}$,*Pank2*$^{fl/fl}$ mice or 3 male and 3 female *SynCre+ Pank1*$^{fl/fl}$,*Pank2*$^{fl/fl}$ mice, and transcript abundance was determined by real-time-qPCR relative to *Gapdh* transcripts in triplicate for each sample. Means ± SEM are plotted. Gray bars are control mice and pink bars are the knockout mice.

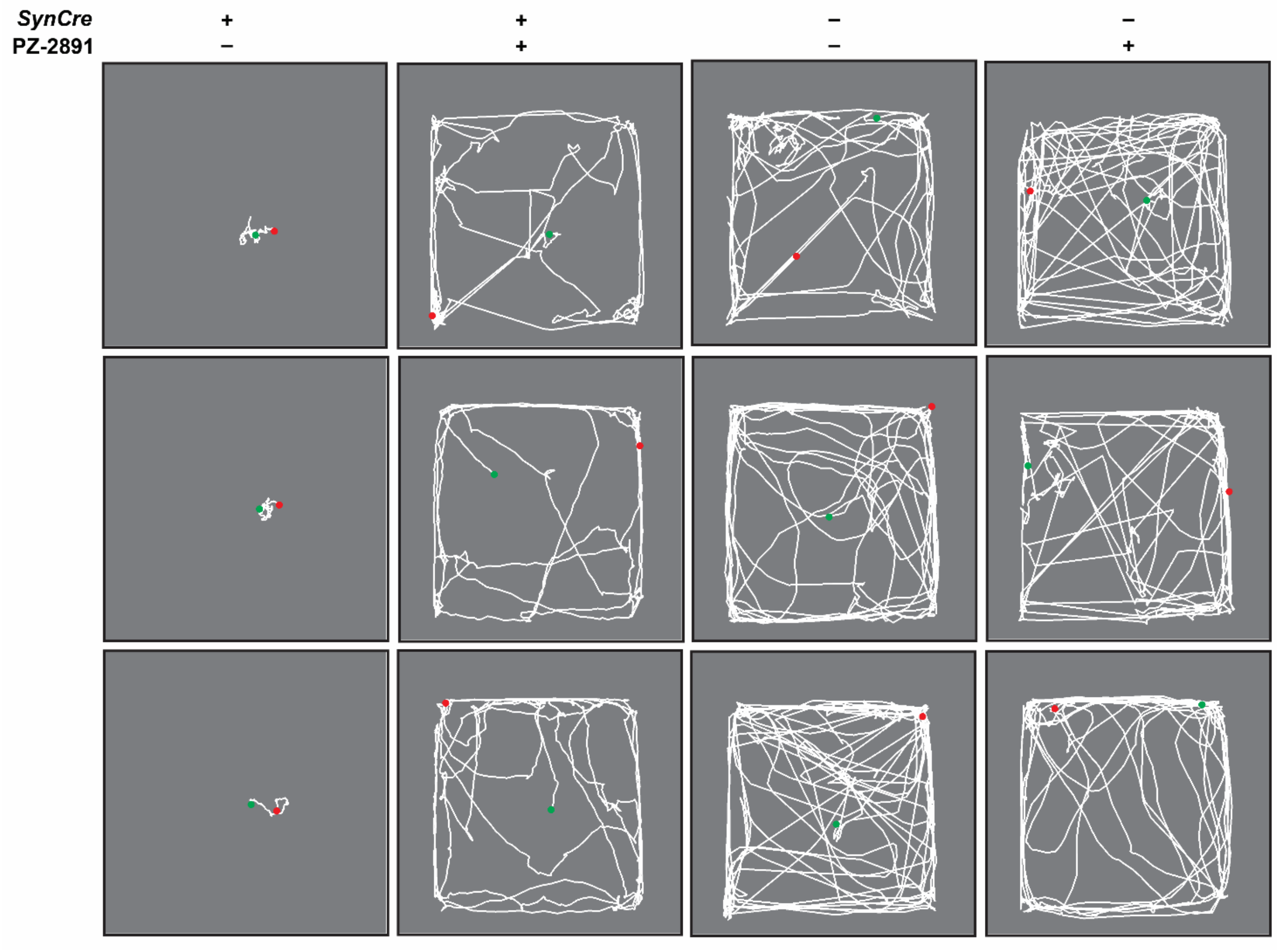


**Supplementary Fig. 16. PZ-2891 restores movement in *Syn-Pank1,Pank2* knockout mice.** Mice were treated, or not, with PZ-2891 and their locomotor activity was scored using the open field test. The green dot shows where the individual animal started and the red dot shows the animal's position at the end of the 5-min observation and tracking period. Three representative examples are shown for each group. (**a**) Untreated *SynCre*+ *Pank1*$^{fl/fl}$,*Pank2*$^{fl/fl}$ neuronal knockout mice. (**b**) *SynCre*+ *Pank1*$^{fl/fl}$,*Pank2*$^{fl/fl}$ mice treated with PZ-2891 for 24 days post-weaning. (**c**) Untreated *SynCre*– *Pank1*$^{fl/fl}$,*Pank2*$^{fl/fl}$ mice. (**d**) *SynCre*– *Pank1*$^{fl/fl}$,*Pank2*$^{fl/fl}$ mice treated with PZ-2891 for 24 days post-weaning. Distance traveled and the percent time in motion were calculated for each animal. These are primary data examples to support the statistical analysis with a larger number of mice shown in Figure 7g, h.

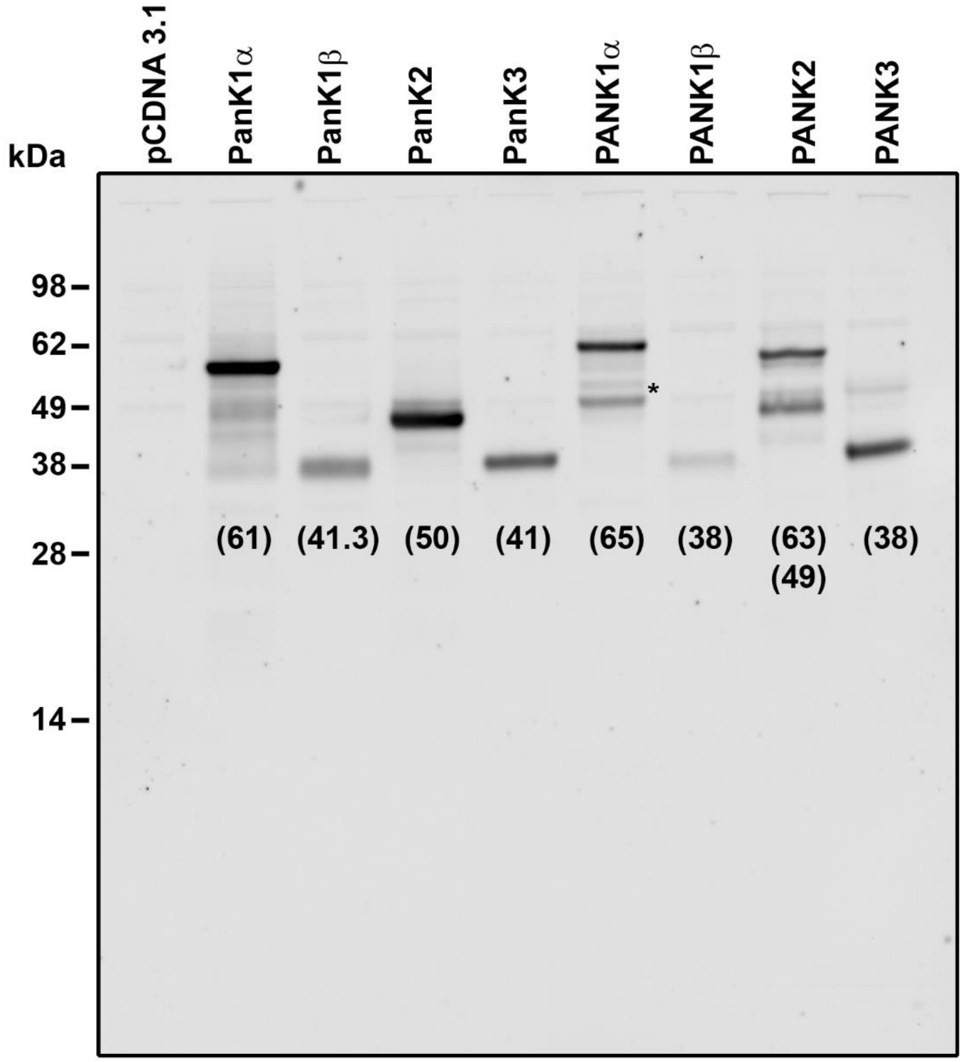


**Supplementary Fig. 17. Validation of α-PANK.** HEK293T cells were transfected with expression vectors for either PanK1α, PanK1β, PanK2, PanK3, PANK1α, PANK1β, PANK2 and PANK3, or the empty vector pcDNA 3.1. 10 μg of total protein was loaded in each lane. The PanK antibody was used at 1:1000 dilution overnight at 4 °C and the secondary antibody was Alexa Fluor 488-goat anti-rabbit IgG (Invitrogen A11008) at 1:2000 dilution. The molecular weights of the proteins in kDa are given in parenthesis in the figure. PANK2 shows both the full length and the processed mature form. The asterisk (*) indicates a non-specific band.

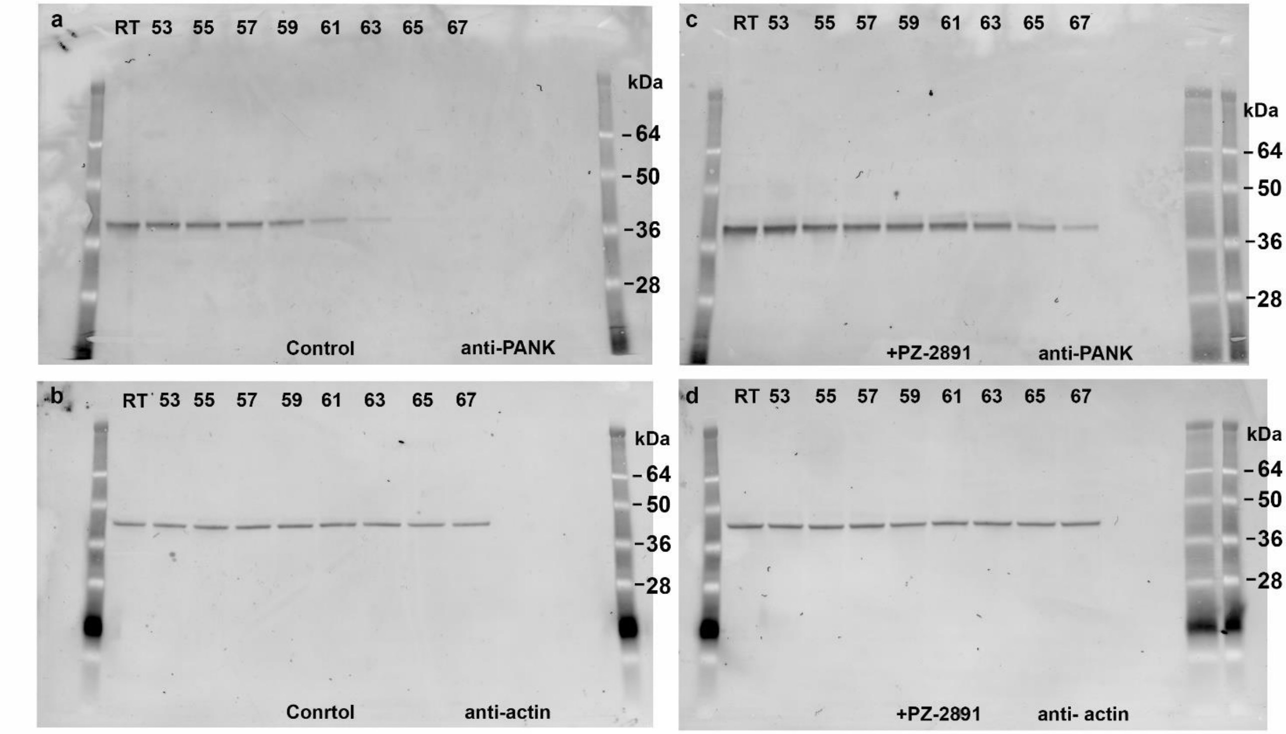


**Supplementary Fig 18. Western Blot for over expression of PANK3 in 293T cells in CETSA.** These data represent the uncropped blots for the cropped image in Fig. 4a. Western blot of cells expressing PANK3 in HEK 293T cells, treated with DMSO or 10 μM PZ-2891, then exposed to increasing temperatures as indicated in the CETSA experiment. (a) Controls treated with DMSO and probed with anti-PANK antibody at 1:1000 dilution overnight at 4°C then probed with Goat anti-rabbit alkaline phosphatase conjugate 1:5000. Bands were detected using the ECF kit (GE Healthcare RPN5783) and visualized by Typhoon phosphor imager. (b) To ensure equal loading the control cells were probed with mouse anti-actin antibody 1:1000 (Sigma A2228) followed by the anti-mouse alkaline phosphatase secondary antibody 1:5000 (GE Healthcare RPN5781) processed as in (a). (c) The cells were treated with 10 μM PZ-2891 for 24 hrs. and the blot was probed with anti-PANK antibody as in (a). (d) To ensure equal loading the cells treated with 10 μM PZ-2891 were blotted and probed as in (b).

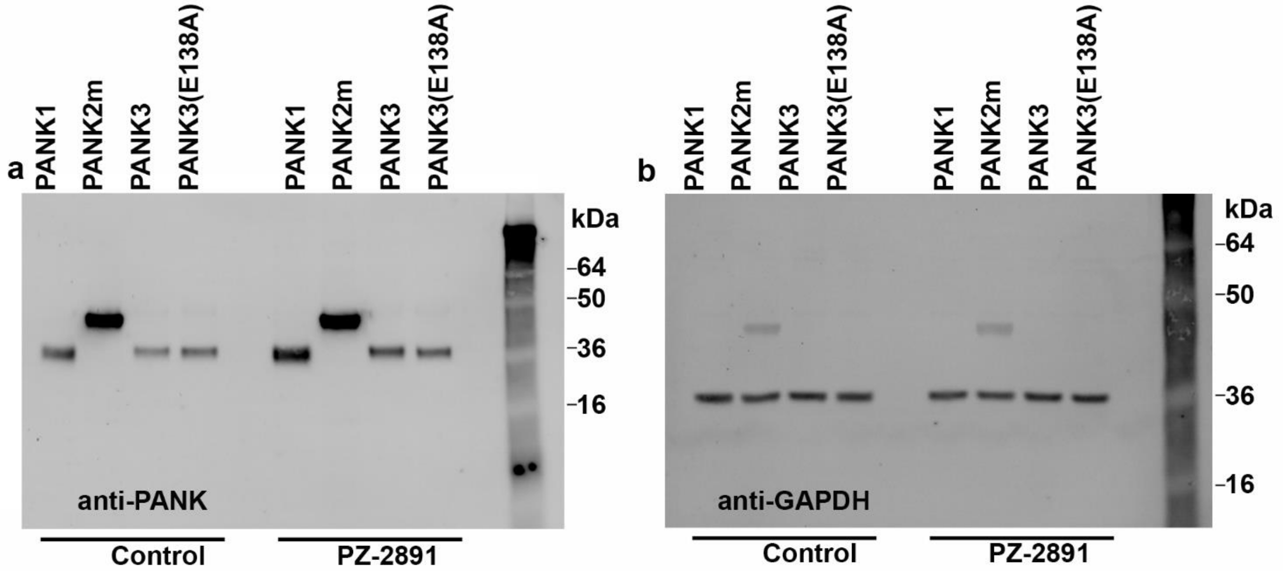


**Supplementary Fig 19. Western Blot for over expression of PANK isoforms in HEK 293T cells.** This data represents the entire blot for the cropped data lacking PANK2m shown in Fig. 4e. (a) Western blot for PANK isoforms in HEK 293T cells probed with anti-PANK antibody. 5 μg of total protein was loaded in each lane. The PANK antibody was used at 1:1000 dilution overnight at 4°C and secondary antibody was Alexa Flour 488-goat anti rabbit IgG (1: 2,000) (Invitrogen A11008). To ensure equal loading 5 μg of the total protein was loaded on another gel and western blotting was performed with anti-GAPDH antibody (1:2500) (Abcam ab9485) and processed as above. The blots were visualized using a Typhoon PhosphorImager.

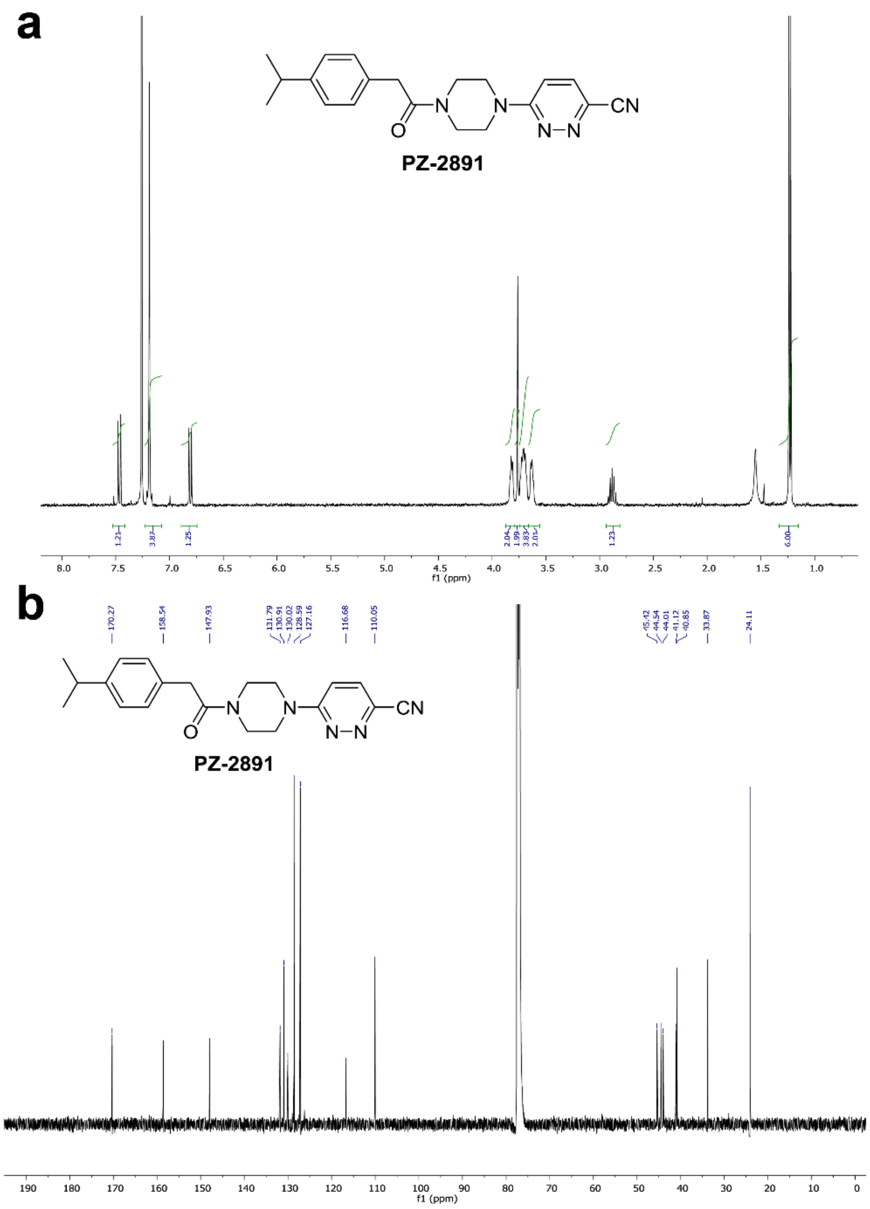


**Supplementary Fig. 20. NMR spectra of PZ-2891.** (**a**) $^{1}H$-NMR spectrum of PZ-2891. (**b**) $^{13}C$-NMR spectrum of PZ-2891. See Methods for details.

**a**

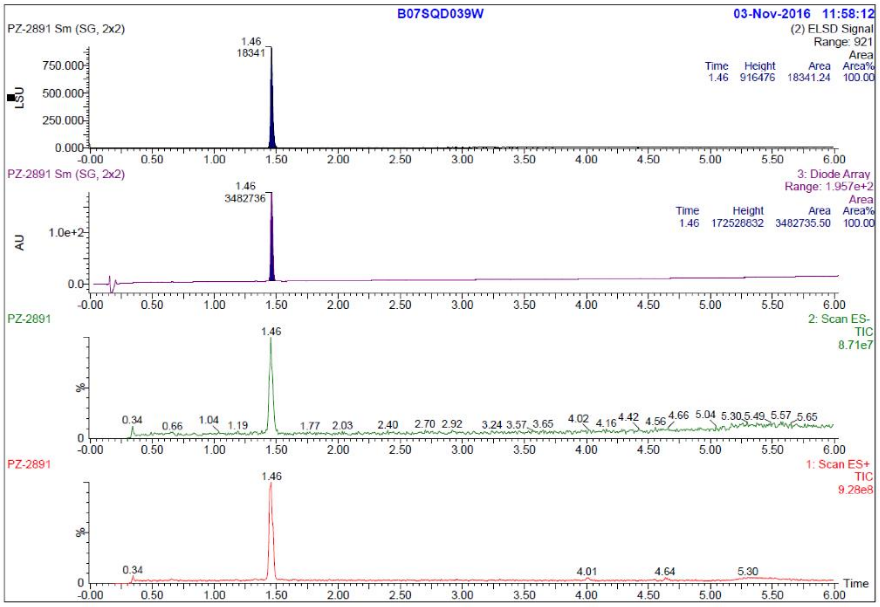


**b**

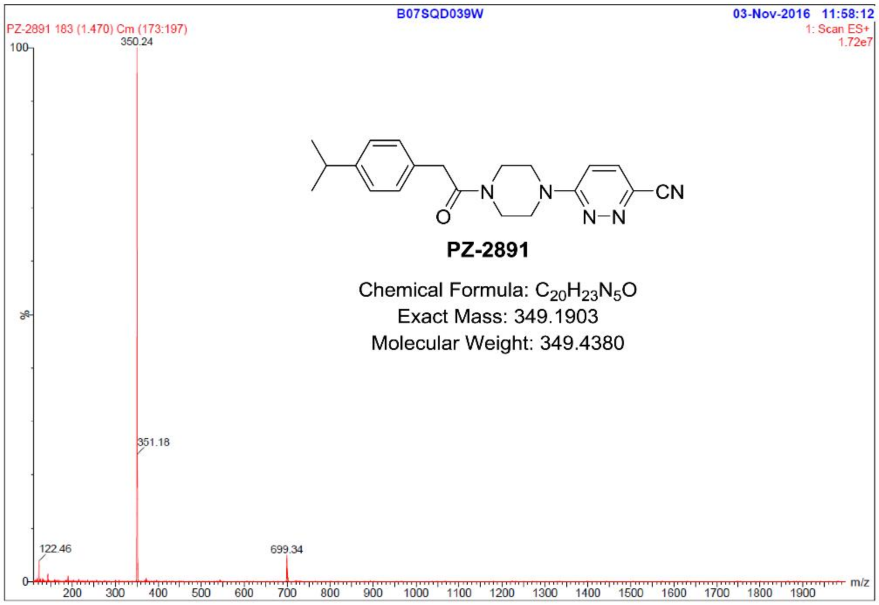


**Supplementary Fig. 21. LC-MS analysis of PZ-2891.** (**a**) Liquid chromatography of PZ-2891. (**b**) Mass spectrum of PZ-2891. See Methods for experimental details.
