# Supplementary material for: A therapeutic approach to pantothenate kinase associated neurodegeneration
Source: Nat Commun. 2018 Oct 23;9:4399. doi: 10.1038/s41467-018-06703-2 (PMC6199309; doi:10.1038/s41467-018-06703-2)

Supplementary Dataset 1. Data supporting graphics presented in Supplementary Figure 7. The first Kinome screen yielded three hits (first DiscoverX primary screen report). These three hits were followed up in a second series of experiments and found to be false positives (second DiscoverX report).

DiscoverX

KINOME*scan*™ Profiling Service

Primary Screen Report

Compound Name	Screening Conc (nM)
PZ-2891	10000

Requester: Lalit K Sharma, Ph.D.

Company: St Jude Childrens Research Hospital (Sharma)

Study Date: 10/11/2016

Report Date: 10/13/2016

Quote ID: MAXXP08486A

Order ID: SMA001-01-p-00001

Product: scanMAX

Number of Targets Tested: 468

Compounds Screened: 1

Jeremy Hunt
Director of Screening
jhunt@discoverx.com
(858) 224-6927 office
(858) 630-4600 fax

LeadHunter™ Discovery Services
DiscoverX Corporation
42501 Albrae Street
Fremont, CA 94538-3142
services.discoverx.com

Technology Overview

The KINOME*scan*™ screening platform employs a novel and proprietary active site-directed competition binding assay to quantitatively measure interactions between test compounds and more than 450 human kinases and disease relevant mutant variants. This robust and reliable assay technology affords investigators the ability to extensively annotate compounds with accurate, precise and reproducible data. KINOME*scan*™ assays do not require ATP and thereby report true thermodynamic interaction affinities, as opposed to IC50 values, which can depend on the ATP concentration.

How KINOME*scan*™ Works

Compounds that bind the kinase active site and directly (sterically) or indirectly (allosterically) prevent kinase binding to the immobilized ligand, will reduce the amount of kinase captured on the solid support (A & B). Conversely, test molecules that do not bind the kinase have no effect on the amount of kinase captured on the solid support (C). Screening "hits" are identified by measuring the amount of kinase captured in test versus control samples by using a quantitative, precise and ultra-sensitive qPCR method that detects the associated DNA label (D). In a similar manner, dissociation constants (Kds) for test compound-kinase interactions are calculated by measuring the amount of kinase captured on the solid support as a function of the test compound concentration.

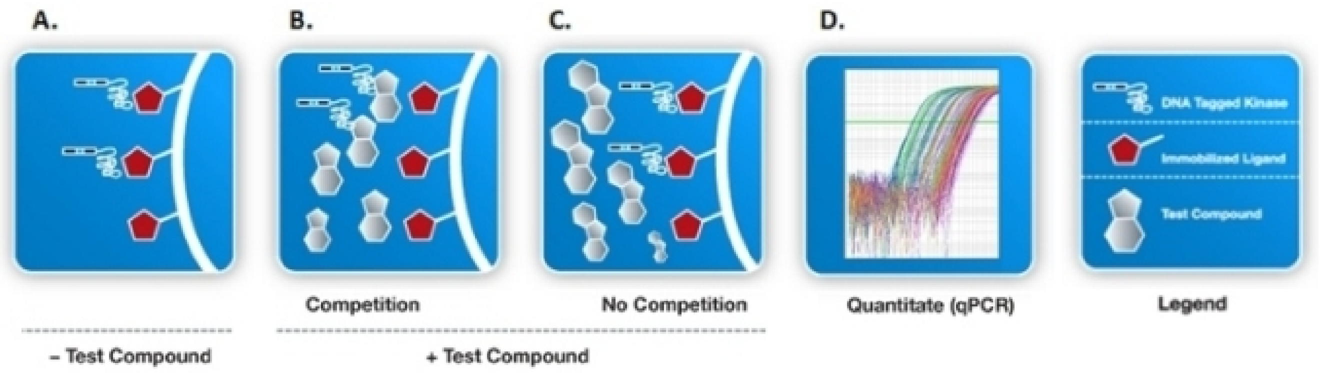

Protocol Description

Kinase assays. For most assays, kinase-tagged T7 phage strains were grown in parallel in 24-well blocks in an *E. coli* host derived from the BL21 strain. *E. coli* were grown to log-phase and infected with T7 phage from a frozen stock (multiplicity of infection = 0.4) and incubated with shaking at 32°C until lysis (90-150 minutes). The lysates were centrifuged (6,000 x g) and filtered (0.2µm) to remove cell debris. The remaining kinases were produced in HEK-293 cells and subsequently tagged with DNA for qPCR detection. Streptavidin-coated magnetic beads were treated with biotinylated small molecule ligands for 30 minutes at room temperature to generate affinity resins for kinase assays. The liganded beads were blocked with excess biotin and washed with blocking buffer (SeaBlock (Pierce), 1 % BSA, 0.05 % Tween 20, 1 mM DTT) to remove unbound ligand and to reduce non-specific phage binding. Binding reactions were assembled by combining kinases, liganded affinity beads, and test compounds in 1x binding buffer (20 % SeaBlock, 0.17x PBS, 0.05 % Tween 20, 6 mM DTT). Test compounds were prepared as 40x stocks in 100% DMSO and directly diluted into the assay. All reactions were performed in polypropylene 384-well plates in a final volume of 0.02 ml. The assay plates were incubated at room temperature with shaking for 1 hour and the affinity beads were washed with wash buffer (1x PBS, 0.05 % Tween 20). The beads were then re-suspended in elution buffer (1x PBS, 0.05 % Tween 20, 0.5 µM non-biotinylated affinity ligand) and incubated at room temperature with shaking for 30 minutes. The kinase concentration in the eluates was measured by qPCR.

Percent Control (%Ctrl)

The compound(s) were screened at the concentration(s) requested, and results for primary screen binding interactions are reported as '% Ctrl', where lower numbers indicate stronger hits in the matrix on the following page(s).

%Ctrl Calculation

$$\left(\frac{\text{test compound signal - positive control signal}}{\text{negative control signal - positive control signal}}\right) \times 100$$

test compound = compound submitted by St Jude Childrens Research Hospital (Sharma)
negative control = DMSO (100%Ctrl)
positive control = control compound (0%Ctrl)

Relationship between Binding Constant Distributions (Kds) &Single Concentration Primary Screen Values

Based on screening data from thousands of profiled compounds, a proportional relationship between primary screening results and corresponding compound/target affinities may be described. Evident in the correlation graph below is a range of binding constants (Kd values) for the indicated ranges of POC values with tighter binding (higher affinity) interactions associated with lower POC values and weaker binding (lower affinity) associated with higher POC values. This distribution of binding constants is characteristic of single concentration primary screens and underscores the importance of following up observed 'hits' or apparent high affinity interactions with quantitative binding constant determinations.

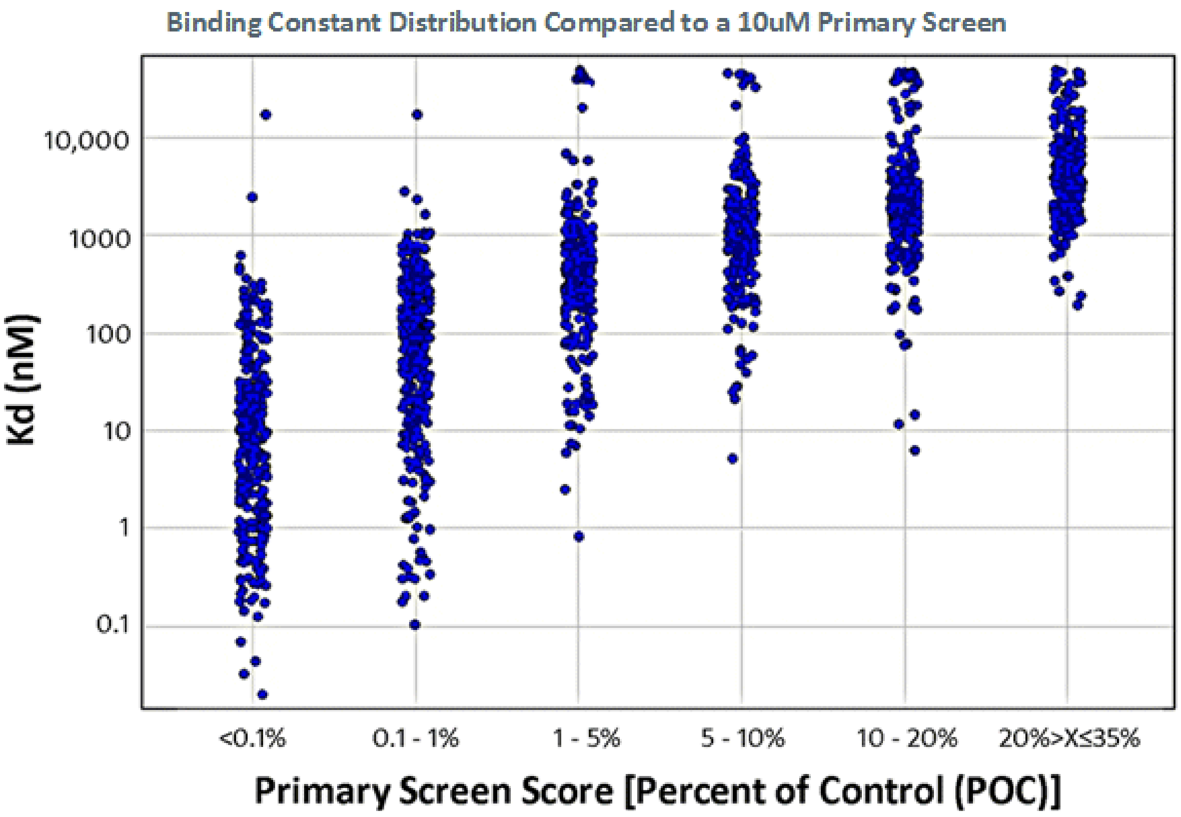

Data correlation between primary screening (10µM concentration) and binding constants (Kd values). Binding constants are correlated with primary screening results, where lower POC values are associated with low Kd values (higher affinity interactions).

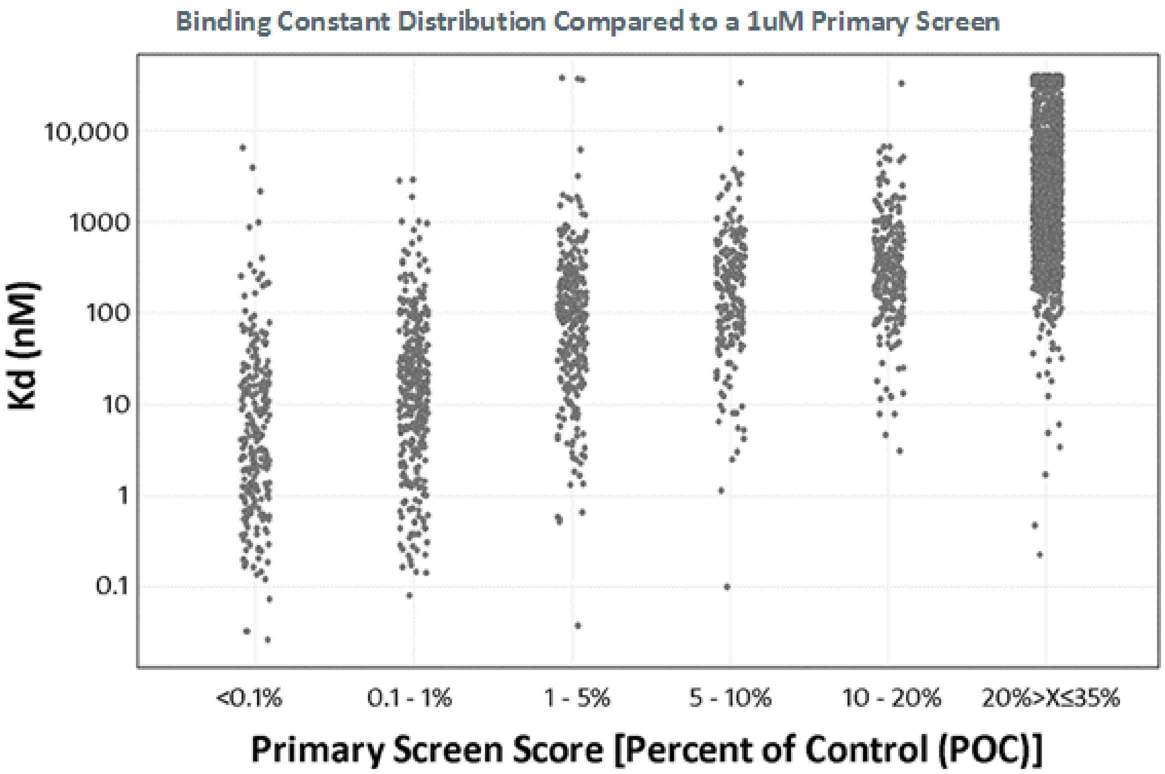

Data correlation between primary screening (1µM concentration) and binding constants (Kd values). Binding constants are correlated with primary screening results, where lower POC values are associated with low Kd values (higher affinity interactions).

Selectivity Score (S-scores)

Selectivity Score or S-score is a quantitative measure of compound selectivity. It is calculated by dividing the number of kinases that compounds bind to by the total number of distinct kinases tested, excluding mutant variants.

S = Number of hits / Number of assays

This value can be calculated using %Ctrl as a potency threshold (below) and provides a quantitative method of describing compound selectivity to facilitate comparison of different compounds.

S(35) = (number of non-mutant kinases with %Ctrl <35)/(number of non-mutant kinases tested)
S(10) = (number of non-mutant kinases with %Ctrl <10)/(number of non-mutant kinases tested)
S(1) = (number of non-mutant kinases with %Ctrl <1)/(number of non-mutant kinases tested)

Using S-Score Data to Quantitate Selectivity

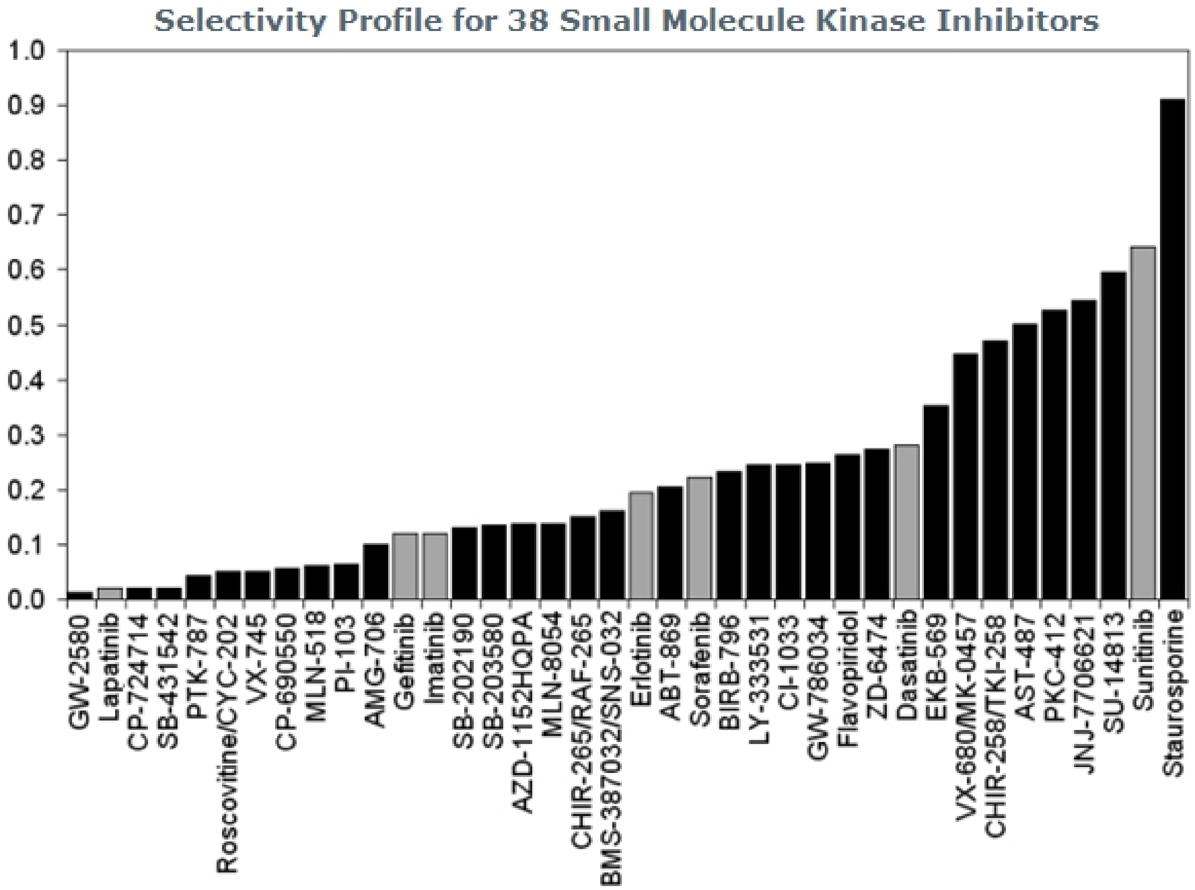

KINOMEscan's in vitro competition binding assay was used to evaluate 38 kinase inhibitors against a panel of 287 distinct human protein kinases (~55% of the predicted human protein kinome), and three lipid kinases. The compounds tested included 21 tyrosine kinase inhibitors, 15 serine-threonine kinase inhibitors, 1 lipid kinase inhibitor and staurosporine. S(35) = (number of non-mutant kinases with %Ctrl <35)/(290 kinases tested; 27 mutant variants were excluded from this analysis). Compounds approved for use in humans (as of August, 2007) are highlighted (gray bars).

References

KINOME*scan*™ and BROMO*scan*™ use the same assay technology. For a more detailed description of this assay technology, see:

• Fabian, M.A. *et al*. A small molecule-kinase interaction map for clinical kinase inhibitors. *Nat. Biotechnol.* 23, 329-336 (2005).

To view kinase interaction maps for 38 well-known kinase inhibitors and a more detailed discussion of selectivity scores, see:

• Karaman, M.W. *et al*. A quantitative analysis of kinase inhibitor selectivity. *Nat. Biotechnol*. 26, 127-132 (2008).

Select publications are available at www.discoverx.com.

SMA001-01-p-00001 Study Results

Table 1 - Matrix of Compound Screen for SMA001-01-p-00001

Target	PZ-2891
Gene Symbol	%Ctrl @ 10000nM
AAK1	84
ABL1(E255K)-phosphorylated	53
ABL1(F317I)-nonphosphorylated	77
ABL1(F317I)-phosphorylated	89
ABL1(F317L)-nonphosphorylated	73
ABL1(F317L)-phosphorylated	100
ABL1(H396P)-nonphosphorylated	74
ABL1(H396P)-phosphorylated	76
ABL1(M351T)-phosphorylated	96
ABL1(Q252H)-nonphosphorylated	97
ABL1(Q252H)-phosphorylated	74
ABL1(T315I)-nonphosphorylated	63
ABL1(T315I)-phosphorylated	97
ABL1(Y253F)-phosphorylated	82
ABL1-nonphosphorylated	65
ABL1-phosphorylated	78
ABL2	96
ACVR1	88
ACVR1B	97
ACVR2A	100
ACVR2B	100
ACVRL1	100
ADCK3	100
ADCK4	92
AKT1	98
AKT2	85
AKT3	82
ALK	100
ALK(C1156Y)	74
ALK(L1196M)	73
AMPK-alpha1	92
AMPK-alpha2	100
ANKK1	78
ARK5	94
ASK1	74
ASK2	83
AURKA	75
AURKB	89
AURKC	94
AXL	73
BIKE	100
BLK	87
BMPR1A	100
BMPR1B	60
BMPR2	87
BMX	100
BRAF	100
BRAF(V600E)	100

DiscoverX

Table 1 - Assay Matrix (continued).

Target	PZ-2891
Gene Symbol	%Ctrl @ 10000nM
BRK	80
BRSK1	75
BRSK2	81
BTK	94
BUB1	100
CAMK1	93
CAMK1B	100
CAMK1D	95
CAMK1G	83
CAMK2A	98
CAMK2B	88
CAMK2D	91
CAMK2G	86
CAMK4	89
CAMKK1	89
CAMKK2	87
CASK	85
CDC2L1	95
CDC2L2	87
CDC2L5	81
CDK11	78
CDK2	87
CDK3	97
CDK4	99
CDK4-cyclinD1	100
CDK4-cyclinD3	71
CDK5	87
CDK7	79
CDK8	83
CDK9	98
CDKL1	74
CDKL2	90
CDKL3	86
CDKL5	67
CHEK1	83
CHEK2	79
CIT	97
CLK1	75
CLK2	94
CLK3	93
CLK4	98
CSF1R	91
CSF1R-autoinhibited	93
CSK	97
CSNK1A1	66
CSNK1A1L	100
CSNK1D	93
CSNK1E	97
CSNK1G1	92

Table 1 - Assay Matrix (continued).

Target	PZ-2891
Gene Symbol	%Ctrl @ 10000nM
CSNK1G2	62
CSNK1G3	80
CSNK2A1	82
CSNK2A2	88
CTK	77
DAPK1	63
DAPK2	95
DAPK3	100
DCAMKL1	80
DCAMKL2	89
DCAMKL3	84
DDR1	81
DDR2	85
DLK	100
DMPK	89
DMPK2	93
DRAK1	99
DRAK2	95
DYRK1A	71
DYRK1B	66
DYRK2	97
EGFR	78
EGFR(E746-A750del)	100
EGFR(G719C)	100
EGFR(G719S)	91
EGFR(L747-E749del, A750P)	72
EGFR(L747-S752del, P753S)	100
EGFR(L747-T751del,Sins)	100
EGFR(L858R)	84
EGFR(L858R,T790M)	98
EGFR(L861Q)	100
EGFR(S752-I759del)	100
EGFR(T790M)	78
EIF2AK1	95
EPHA1	95
EPHA2	100
EPHA3	65
EPHA4	93
EPHA5	88
EPHA6	89
EPHA7	92
EPHA8	95
EPHB1	90
EPHB2	82
EPHB3	92
EPHB4	97
EPHB6	68
ERBB2	92
ERBB3	60

DiscoverX

Table 1 - Assay Matrix (continued).

Target	PZ-2891
Gene Symbol	%Ctrl @ 10000nM
ERBB4	100
ERK1	91
ERK2	86
ERK3	97
ERK4	97
ERK5	93
ERK8	81
ERN1	82
FAK	89
FER	88
FES	94
FGFR1	87
FGFR2	100
FGFR3	96
FGFR3(G697C)	99
FGFR4	73
FGR	96
FLT1	100
FLT3	57
FLT3(D835H)	92
FLT3(D835V)	78
FLT3(D835Y)	89
FLT3(ITD)	99
FLT3(ITD,D835V)	85
FLT3(ITD,F691L)	76
FLT3(K663Q)	88
FLT3(N841I)	86
FLT3(R834Q)	100
FLT3-autoinhibited	82
FLT4	91
FRK	86
FYN	87
GAK	93
GCN2(Kin.Dom.2,S808G)	87
GRK1	72
GRK2	56
GRK3	35
GRK4	52
GRK7	93
GSK3A	83
GSK3B	74
HASPIN	85
HCK	79
HIPK1	61
HIPK2	100
HIPK3	81
HIPK4	94
HPK1	100
HUNK	100

DiscoverX

Table 1 - Assay Matrix (continued).

Target	PZ-2891
Gene Symbol	%Ctrl @ 10000nM
ICK	81
IGF1R	91
IKK-alpha	100
IKK-beta	100
IKK-epsilon	89
INSR	84
INSRR	82
IRAK1	91
IRAK3	42
IRAK4	93
ITK	89
JAK1(JH1domain-catalytic)	74
JAK1(JH2domain-pseudokinase)	47
JAK2(JH1domain-catalytic)	88
JAK3(JH1domain-catalytic)	94
JNK1	62
JNK2	79
JNK3	88
KIT	75
KIT(A829P)	91
KIT(D816H)	66
KIT(D816V)	92
KIT(L576P)	88
KIT(V559D)	81
KIT(V559D,T670I)	94
KIT(V559D,V654A)	73
KIT-autoinhibited	81
LATS1	86
LATS2	82
LCK	98
LIMK1	87
LIMK2	95
LKB1	93
LOK	86
LRRK2	100
LRRK2(G2019S)	100
LTK	75
LYN	79
LZK	89
MAK	69
MAP3K1	89
MAP3K15	92
MAP3K2	96
MAP3K3	65
MAP3K4	93
MAP4K2	77
MAP4K3	94
MAP4K4	100
MAP4K5	100

Table 1 - Assay Matrix (continued).

Target	PZ-2891
Gene Symbol	%Ctrl @ 10000nM
MAPKAPK2	88
MAPKAPK5	74
MARK1	77
MARK2	70
MARK3	84
MARK4	81
MAST1	61
MEK1	76
MEK2	80
MEK3	61
MEK4	100
MEK5	69
MEK6	94
MELK	75
MERTK	95
MET	80
MET(M1250T)	98
MET(Y1235D)	66
MINK	74
MKK7	84
MKNK1	97
MKNK2	79
MLCK	100
MLK1	81
MLK2	72
MLK3	80
MRCKA	92
MRCKB	100
MST1	86
MST1R	73
MST2	92
MST3	73
MST4	91
MTOR	68
MUSK	95
MYLK	75
MYLK2	98
MYLK4	92
MYO3A	68
MYO3B	88
NDR1	75
NDR2	94
NEK1	100
NEK10	89
NEK11	99
NEK2	88
NEK3	69
NEK4	95
NEK5	100

Table 1 - Assay Matrix (continued).

Target	PZ-2891
Gene Symbol	%Ctrl @ 10000nM
NEK6	91
NEK7	75
NEK9	96
NIK	100
NIM1	83
NLK	94
OSR1	87
p38-alpha	100
p38-beta	96
p38-delta	76
p38-gamma	58
PAK1	76
PAK2	44
PAK3	88
PAK4	92
PAK6	100
PAK7	73
PCTK1	76
PCTK2	95
PCTK3	92
PDGFRA	70
PDGFRB	75
PDPK1	75
PFCDPK1(P.falciparum)	97
PFPK5(P.falciparum)	99
PFTAIRE2	99
PFTK1	92
PHKG1	88
PHKG2	100
PIK3C2B	100
PIK3C2G	61
PIK3CA	95
PIK3CA(C420R)	76
PIK3CA(E542K)	64
PIK3CA(E545A)	64
PIK3CA(E545K)	65
PIK3CA(H1047L)	69
PIK3CA(H1047Y)	46
PIK3CA(I800L)	42
PIK3CA(M1043I)	74
PIK3CA(Q546K)	70
PIK3CB	65
PIK3CD	89
PIK3CG	96
PIK4CB	84
PIKFYVE	72
PIM1	93
PIM2	96
PIM3	58

Table 1 - Assay Matrix (continued).

Target	PZ-2891
Gene Symbol	%Ctrl @ 10000nM
PIP5K1A	86
PIP5K1C	100
PIP5K2B	88
PIP5K2C	85
PKAC-alpha	87
PKAC-beta	95
PKMYT1	100
PKN1	69
PKN2	94
PKNB(M.tuberculosis)	74
PLK1	85
PLK2	84
PLK3	78
PLK4	88
PRKCD	89
PRKCE	70
PRKCH	92
PRKCI	83
PRKCQ	100
PRKD1	89
PRKD2	82
PRKD3	66
PRKG1	99
PRKG2	76
PRKR	100
PRKX	70
PRP4	86
PYK2	90
QSK	82
RAF1	85
RET	74
RET(M918T)	95
RET(V804L)	96
RET(V804M)	95
RIOK1	100
RIOK2	86
RIOK3	100
RIPK1	98
RIPK2	89
RIPK4	81
RIPK5	77
ROCK1	100
ROCK2	100
ROS1	81
RPS6KA4(Kin.Dom.1-N-terminal)	96
RPS6KA4(Kin.Dom.2-C-terminal)	67
RPS6KA5(Kin.Dom.1-N-terminal)	93
RPS6KA5(Kin.Dom.2-C-terminal)	96
RSK1(Kin.Dom.1-N-terminal)	100

Table 1 - Assay Matrix (continued).

Target	PZ-2891
Gene Symbol	%Ctrl @ 10000nM
RSK1(Kin.Dom.2-C-terminal)	64
RSK2(Kin.Dom.1-N-terminal)	80
RSK2(Kin.Dom.2-C-terminal)	89
RSK3(Kin.Dom.1-N-terminal)	72
RSK3(Kin.Dom.2-C-terminal)	66
RSK4(Kin.Dom.1-N-terminal)	97
RSK4(Kin.Dom.2-C-terminal)	71
S6K1	96
SBK1	62
SGK	61
SgK110	94
SGK2	64
SGK3	71
SIK	83
SIK2	9.5
SLK	93
SNARK	69
SNRK	85
SRC	97
SRMS	72
SRPK1	100
SRPK2	97
SRPK3	67
STK16	98
STK33	90
STK35	0.2
STK36	84
STK39	70
SYK	82
TAK1	67
TAOK1	93
TAOK2	99
TAOK3	95
TBK1	60
TEC	79
TESK1	86
TGFBR1	99
TGFBR2	98
TIE1	85
TIE2	97
TLK1	72
TLK2	98
TNIK	91
TNK1	100
TNK2	98
TNNI3K	76
TRKA	100
TRKB	100
TRKC	100

Table 1 - Assay Matrix (continued).

Target	PZ-2891
Gene Symbol	%Ctrl @ 10000nM
TRPM6	98
TSSK1B	99
TSSK3	69
TTK	96
TXK	92
TYK2(JH1domain-catalytic)	67
TYK2(JH2domain-pseudokinase)	100
TYRO3	100
ULK1	82
ULK2	86
ULK3	89
VEGFR2	75
VPS34	80
VRK2	70
WEE1	92
WEE2	99
WNK1	100
WNK2	87
WNK3	100
WNK4	69
YANK1	74
YANK2	100
YANK3	84
YES	90
YSK1	100
YSK4	89
ZAK	88
ZAP70	96

%Ctrl Legend

0≤x<.1	.1≤x<1	1≤x<10	10≤x<35	x≥35

S-score Results

Table 2 - S-score Table for SMA001-01-p-00001

Compound Name	Selectivity Score Type	Number of Hits	Number of Non-Mutant Kinases	Screening Concentration (nM)	Selectivity Score
PZ-2891	S(35)	2	403	10000	0.005
PZ-2891	S(10)	2	403	10000	0.005
PZ-2891	S(1)	1	403	10000	0.002

TREE*spot*™ Interaction Maps - Now Includes Mutant, Lipid, Atypical & Pathogen Kinase Dendrograms

As part of our ongoing effort to provide customers with the best possible data analysis tools, KINOME*scan*™ has developed an enhanced rendering of the human kinase dendrogram and allows, for the first time ever, to fully visualize compound interactions across our industry leading kinase panel, including clinically and biochemically relevant mutants, lipid, atypical, and pathogen kinases, plus a growing panel of activation-state specific assays.

TREE*spot*™ is an artistic representation of the human kinome phylogenetic tree based on extensive published research. We welcome your comments and feedback on this new visualization image. Please contact us at sales@kinomescan.com to tell us what you think.

Key Changes

- More uniform format and presentation
- Kinase groups more clearly delineated
- Updated nomenclature for kinases

TREE*spot*™ is a proprietary data visualization software tool developed by KINOME*scan*. *Mutant and lipid kinases are not represented.* Kinases found to bind are marked with red circles, where larger circles indicate higher-affinity binding. Visualize data online and create your own high resolution TREE*spot*™ interaction maps with our easy-to-use compound profile visualization tool. Instructions and login credentials provided below.

Login: treespot.discoverx.com -- **Username:** treespot! -- **Password:** guest037
Instructions: treespot.discoverx.com/Help/TreeSpotHelpBasic.htm

Table 3 - TREE*spot*™ Interaction Maps for SMA001-01-p-00001

PZ-2891 @ 10000nM

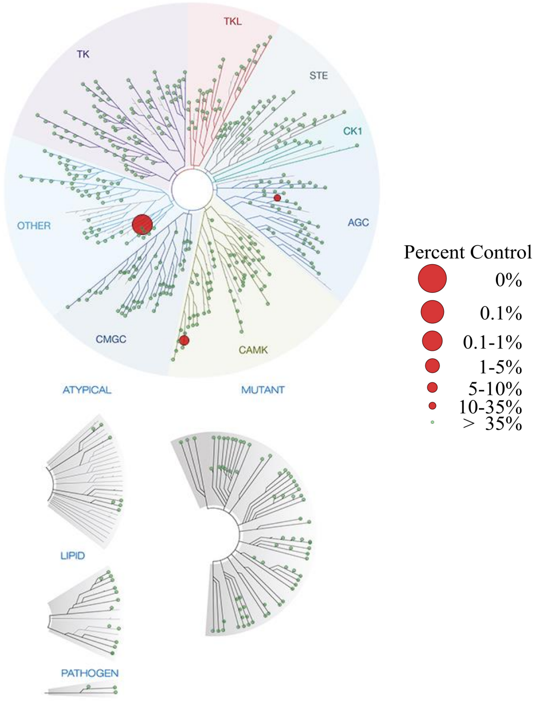

Available Follow-up Screening Services

LeadHunter™ Discovery Services offers a suite of investigative tools that enable detailed biochemical characterization of the interaction between inhibitors and their targets. The thermodynamic, kinetic, and structural information provided by these tools enables a detailed comparison of inhibitors from common or distinct lead series and facilitates the interpretation of data from downstream cellular and *in vivo* pharmacology models. These services are now available for both kinases and for bromodomain-containing proteins.

KdELECT Obtain quantitative binding affinities for compound-kinase interactions

***Kd*ELECT™** - a powerful follow up service to quantify binding affinity of compound-kinase interactions identified in primary (single concentration) screens. Inhibitor binding constants (Kd values) are calculated from duplicate 11-point dose-response curves under optimized conditions that generate true thermodynamic Kd values which facilitate direct comparison of inhibitor affinity across kinases. Learn more >>

Now Available! PathHunter® cell-based compound screening & profiling services

PathHunter inCell assays and screening services are a powerful follow up solution to KINOME*scan*™ *in vitro* biochemical studies for obtaining the maximum level of information about inhibitor function, potency and selectivity in a more physiological context.

DiscoverX

KINOMEscan™ Profiling Service

Kd Report

Compound Name	Kds Determined
PZ-2891	3

Requester: Lalit K Sharma, Ph.D.

Company: St Jude Childrens Research Hospital (Sharma)

Study Date: 10/27/2016

Report Date: 10/27/2016

Quote ID: KDELS04303A

Order ID: SMA003-01-s-00001

Product: KdELECT

Number of Kds Determined: 3

Jeremy Hunt
Director of Screening
jhunt@discoverx.com
(858) 224-6927 office
(858) 630-4600 fax

LeadHunter™ Discovery Services
DiscoverX Corporation
42501 Albrae Street
Fremont, CA 94538-3142
services.discoverx.com

Technology Overview

KINOME*scan*™ is the industry's most comprehensive high-throughput system for screening compounds against large numbers of human kinases. Developed by DiscoverX, KINOME*scan*™ employs proprietary active-site dependent competition binding assays to determine how compounds bind to both intended and unintended kinases. In addition to helping keep discovery programs on track, KINOME*scan*™ can opportunistically identify unanticipated interactions that can expand the therapeutic utility of compounds or serve as advanced starting points for new programs.

How KINOME***scan*™** Works

KINOME*scan*™ is based on a competition binding assay that quantitatively measures the ability of a compound to compete with an immobilized, active-site directed ligand. The assay is performed by combining three components: DNA-tagged kinase; immobilized ligand; and a test compound. The ability of the test compound to compete with the immobilized ligand is measured via quantitative PCR of the DNA tag.

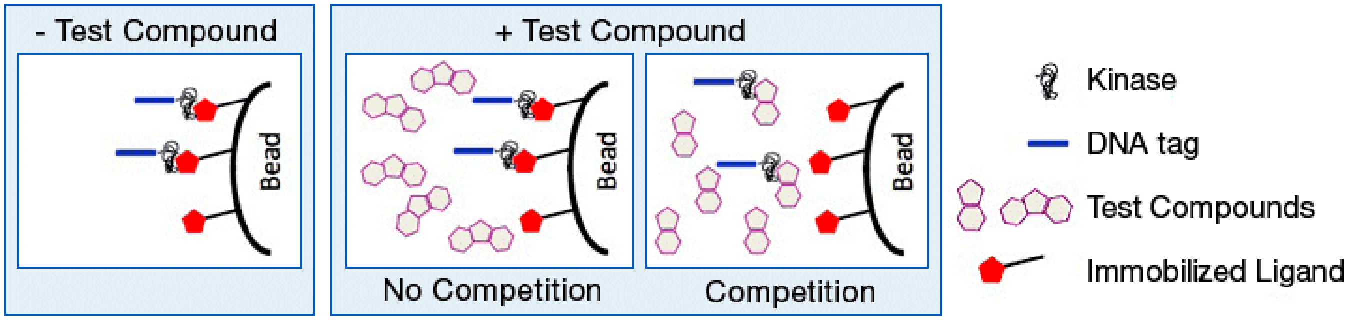

Protocol Description

Kinase assays. For most assays, kinase-tagged T7 phage strains were prepared in an *E. coli* host derived from the BL21 strain. *E. coli* were grown to log-phase and infected with T7 phage and incubated with shaking at 32°C until lysis. The lysates were centrifuged and filtered to remove cell debris. The remaining kinases were produced in HEK-293 cells and subsequently tagged with DNA for qPCR detection. Streptavidin-coated magnetic beads were treated with biotinylated small molecule ligands for 30 minutes at room temperature to generate affinity resins for kinase assays. The liganded beads were blocked with excess biotin and washed with blocking buffer (SeaBlock (Pierce), 1% BSA, 0.05% Tween 20, 1 mM DTT) to remove unbound ligand and to reduce non-specific binding. Binding reactions were assembled by combining kinases, liganded affinity beads, and test compounds in 1x binding buffer (20% SeaBlock, 0.17x PBS, 0.05% Tween 20, 6 mM DTT). Test compounds were prepared as 111X stocks in 100% DMSO. Kds were determined using an 11-point 3-fold compound dilution series with three DMSO control points. All compounds for Kd measurements are distributed by acoustic transfer (non-contact dispensing) in 100% DMSO. The compounds were then diluted directly into the assays such that the final concentration of DMSO was 0.9%. All reactions performed in polypropylene 384-well plate. Each was a final volume of 0.02 ml. The assay plates were incubated at room temperature with shaking for 1 hour and the affinity beads were washed with wash buffer (1x PBS, 0.05% Tween 20). The beads were then re-suspended in elution buffer (1x PBS, 0.05% Tween 20, 0.5 µM non-biotinylated affinity ligand) and incubated at room temperature with shaking for 30 minutes. The kinase concentration in the eluates was measured by qPCR.

Compound Handling

An 11-point 3-fold serial dilution of each test compound was prepared in 100% DMSO at 100x final test concentration and subsequently diluted to 1x in the assay (final DMSO concentration = 1%). Most Kds were determined using a compound top concentration = 30,000 nM. If the initial Kd determined was < 0.5 nM (the lowest concentration tested), the measurement was repeated with a serial dilution starting at a lower top concentration. A Kd value reported as 40,000 nM indicates that the Kd was determined to be >30,000 nM.

Binding Constants (Kds)

Binding constants (Kds) were calculated with a standard dose-response curve using the Hill equation:

$$\text{Response} = \text{Background} + \frac{\text{Signal} - \text{Background}}{1 + (\text{Kd}^{\text{Hill Slope}} / \text{Dose}^{\text{Hill Slope}})}$$

The Hill Slope was set to -1.

Curves were fitted using a non-linear least square fit with the Levenberg-Marquardt algorithm.

TREE*spot*™ Compound Profile Visualization Tool

TREE*spot*™ is a proprietary data visualization software tool developed by KINOME*scan*™. Visualize data online and create your own high resolution TREE*spot*™ interaction maps with our easy-to-use compound profile visualization tool. TREE*spot*™ is provided as a complimentary service to our clients. To access TREE*spot*™, please follow these directions:

Login: treespot.discoverx.com -- Username: treespot! -- Password: guest037
Instructions: treespot.discoverx.com/Help/TreeSpotHelpBasic.htm

References

KINOME*scan*™ and BROMO*scan*™ use the same assay technology. For a more detailed description of this assay technology, see:

- Fabian, M.A. *et al*. A small molecule-kinase interaction map for clinical kinase inhibitors. *Nat. Biotechnol.* 23, 329-336 (2005).

To view kinase interactions for 38 well-known kinase inhibitors, see:

- Karaman, M.W. *et al*. A quantitative analysis of kinase inhibitor selectivity. *Nat. Biotechnol.* 26, 127-132 (2008).

For examples on how KINOME*scan* can opportunistically identify unanticipated therapeutically-beneficial interactions, see:

- Carter, T.A. *et al*. Inhibition of drug-resistant mutants of ABL, KIT, and EGF receptor kinases. *Proc. Natl. Acad. Sci.* USA. 102, 11011-11016 (2005)

For more information on the Hill equation and the Levenberg-Marquardt algorithm, see:

- Hill, A. V. The possible effects of the aggregation of the molecules of hemoglobin on its dissociation curves. *J. Physiol. (Lond.).* 40, iv-vii (1910).
- Levenberg, K. A method for the solution of certain non-linear problems in least squares. *Q. Appl. Math.* 2, 164-168 (1944).

Select publications are available at www.discoverx.com.

DiscoverX

SMA003-01-s-00001 Study Results

Table 1 - Matrix of Kds for SMA003-01-s-00001.

Target	PZ-2891
Gene Symbol	Kd (nM)
GRK3	>30000
SIK2	>30000
STK35	>30000

Kd Legend

x<100nM	100nM≤x<1uM	x≥1uM	No Binding	Not Requested

SMA003-01-s-00001 Curve Images

Table 2 - Curve Images for SMA003-01-s-00001. The amount of kinase measured by qPCR (Signal; y-axis) is plotted against the corresponding compound concentration in nM in log10 scale (x-axis). Data points marked with an "x" were not used for Kd determination.

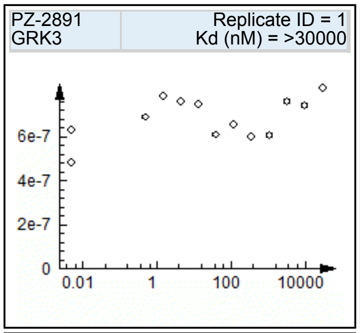

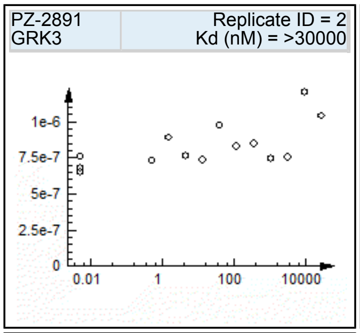

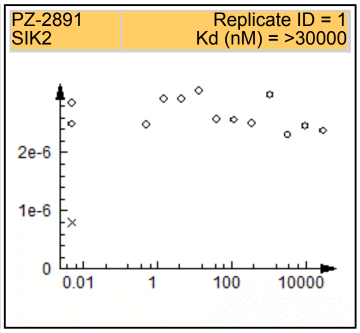

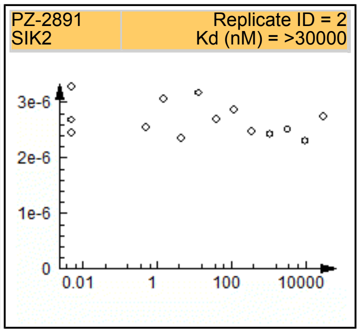

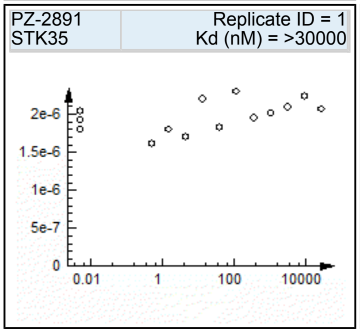

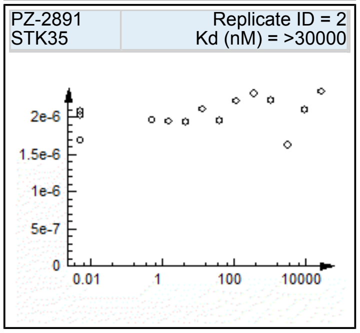

Supplement: Supplementary file 3 — Supplementary Dataset 1 [file 41467_2018_6703_MOESM3_ESM.pdf]
