# Supplementary material for: A therapeutic approach to pantothenate kinase associated neurodegeneration
Source: Nat Commun. 2018 Oct 23;9:4399. doi: 10.1038/s41467-018-06703-2 (PMC6199309; doi:10.1038/s41467-018-06703-2)
Supplement: Supplementary file 4 — Supplementary Dataset 2 [file 41467_2018_6703_MOESM4_ESM.pdf]

DiscoverX Gene Symbol	Assay Mode	PZ-2891 1 μM % Response			PZ-2891 10 μM % Response		
**GPCRs**		Replicate 1	Replicate 2	Average	Replicate 1	Replicate 2	Average
ADORA2A	Agonist	-14.5	-16.2	-15.4	-19.5	-6.0	-12.8
ADORA2A	Antagonist	-7.4	-13.6	-10.5	-6.4	9.5	1.6
ADRA1A	Agonist	-23.7	-25.0	-24.4	-24.6	-24.5	-24.6
ADRA1A	Antagonist	-9.2	-6.5	-7.9	0.6	12.4	6.5
ADRA2A	Agonist	10.5	4.3	7.4	11.2	3.8	7.5
ADRA2A	Antagonist	-4.9	-3.0	-4.0	-5.1	-7.8	-6.5
ADRB1	Agonist	1.1	0.9	1.0	1.0	0.1	0.6
ADRB1	Antagonist	7.6	1.3	4.5	11.6	8.6	10.1
ADRB2	Agonist	0.7	0.1	0.4	0.2	0.2	0.2
ADRB2	Antagonist	8.4	9.8	9.1	27.0	23.8	25.4
AVPR1A	Agonist	-0.8	-0.5	-0.7	-0.9	-1.2	-1.1
AVPR1A	Antagonist	0.8	13.2	7.0	8.0	29.0	18.5
CCKAR	Agonist	-5.2	-4.7	-5.0	-4.5	-4.6	-4.6
CCKAR	Antagonist	0.9	3.1	2.0	14.6	8.6	11.6
CHRM1	Agonist	-0.4	-0.8	-0.6	-2.2	-1.9	-2.1
CHRM1	Antagonist	1.3	1.5	1.4	13.5	13.1	13.3
CHRM2	Agonist	9.8	15.8	12.8	29.5	40.6	35.1
CHRM2	Antagonist	-7.4	-6.2	-6.8	-7.9	-13.3	-10.6
CHRM3	Agonist	-1.3	-3.3	-2.3	-4.2	-2.7	-3.5
CHRM3	Antagonist	1.6	9.3	5.5	6.1	12.3	9.2
CNR1	Agonist	11.8	15.5	13.7	42.3	38.7	40.5
CNR1	Antagonist	-2.9	-1.6	-2.3	-1.6	-5.6	-3.6
CNR2	Agonist	2.7	13.8	8.3	10.3	4.2	7.3
CNR2	Antagonist	1.4	-0.1	0.7	-0.7	-1.8	-1.3
DRD1	Agonist	1.1	0.6	0.9	-0.6	0.6	0.0
DRD1	Antagonist	32.9	32.9	32.9	51.3	53.3	52.3
DRD2S	Agonist	8.9	21.9	15.4	16.7	21.6	19.2
DRD2S	Antagonist	-0.8	-0.4	-0.6	-1.1	-1.2	-1.2
EDNRA	Agonist	-2.7	-3.3	-3.0	-2.6	-2.5	-2.6
EDNRA	Antagonist	2.9	0.5	1.7	5.1	7.9	6.5
HRH1	Agonist	-0.8	-0.6	-0.7	-0.9	-0.8	-0.9
HRH1	Antagonist	5.6	1.4	3.5	7.1	3.0	5.1
HRH2	Agonist	0.2	0.0	0.1	-0.5	-0.2	-0.4
HRH2	Antagonist	1.5	4.5	3.0	15.3	18.8	17.1
HTR1A	Agonist	14.8	10.9	12.9	10.1	11.4	10.8
HTR1A	Antagonist	-0.7	-4.8	-2.8	-3.6	-3.0	-3.3
HTR1B	Agonist	9.5	14.7	12.1	12.3	15.0	13.7
HTR1B	Antagonist	-0.5	-0.2	-0.4	-1.1	-0.8	-1.0
HTR2A	Agonist	3.0	-5.9	-1.5	-2.3	-1.8	-2.1
HTR2A	Antagonist	1.6	7.3	4.5	14.8	14.4	14.6
HTR2B	Agonist	8.0	9.6	8.8	12.4	7.4	9.9
HTR2B	Antagonist	17.4	5.6	11.5	28.1	47.5	37.8
OPRD1	Agonist	14.7	18.2	16.5	21.7	17.5	19.6
OPRD1	Antagonist	0.0	0.2	0.1	0.2	-1.3	-0.6
OPRK1	Agonist	-1.9	-12.2	-7.1	31.8	33.6	32.7
OPRK1	Antagonist	-1.3	0.0	-0.7	-1.2	-0.3	-0.8
OPRM1	Agonist	-1.1	3.3	1.1	11.0	14.1	12.6
OPRM1	Antagonist	-0.7	-1.3	-1.0	-2.0	-1.8	-1.9

DiscoverX Gene Symbol	Assay Mode	PZ-2891 1 μM % Response			PZ-2891 10 μM % Response		
**Nuclear Hormone Receptors**		Replicate 1	Replicate 2	Average	Replicate 1	Replicate 2	Average
AR	Agonist	-0.5	-0.5	-0.5	-0.5	-0.5	-0.5
AR	Antagonist	23.4	17.1	20.3	15.9	-3.1	6.4
GR	Agonist	-0.9	0.9	0.0	0.1	0.5	0.3
GR	Antagonist	8.8	-11.1	-1.2	4.9	-15.7	-5.4
**Transporters**		Replicate 1	Replicate 2	Average	Replicate 1	Replicate 2	Average
DAT	Blocker	-13.1	9.6	-1.8	-3.8	-7.1	-5.5
NET	Blocker	22.4	18.6	20.5	20.1	19.5	19.8
SERT	Blocker	2.0	1.0	1.5	3.2	7.3	5.3
**Ion Channels**		Replicate 1	Replicate 2	Average	Replicate 1	Replicate 2	Average
CAV1.2	Blocker	0.6	-0.6	0.0	7.7	5.9	6.8
GABAA	Opener	1.7	0.8	1.3	0.0	1.1	0.6
GABAA	Blocker	-13.2	-24.1	-18.7	-19.3	-25.7	-22.5
hERG	Blocker	-36.2	-1.4	-18.8	7.2	-18.4	-5.6
HTR3A	Opener	-10.4	-18.0	-14.2	-2.3	-19.7	-11.0
HTR3A	Blocker	-7.3	-21.6	-14.5	4.8	4.4	4.6
NAV1.5	Blocker	-22.1	-16.8	-19.5	-19.6	-15.5	-17.6
**Non-Kinase Enzymes**		Replicate 1	Replicate 2	Average	Replicate 1	Replicate 2	Average
AChE	Inhibitor	9.1	-0.1	4.5	22.2	17.0	19.6
COX1	Inhibitor	-2.4	-7.1	-4.7	13.6	10.1	11.8
COX2	Inhibitor	4.2	-1.3	1.5	23.4	21.6	22.5
MAOA	Inhibitor	-0.8	-4.7	-2.8	4.6	2.6	3.6
PDE3A	Inhibitor	16.6	16.6	16.6	65.4	66.8	66.1
PDE4D2	Inhibitor	2.3	-2.3	0.0	0.0	0.0	0.0
**Kinases**		Replicate 1	Replicate 2	Average	Replicate 1	Replicate 2	Average
INSR	Inhibitor	10.7	0.7	5.7	-3.3	16.0	6.4
LCK	Inhibitor	1.8	-18.4	-8.3	-12.4	-3.6	-8.0
ROCK1	Inhibitor	-12.3	-8.5	-10.4	8.5	6.9	7.7
VEGFR2	Inhibitor	-8.2	0.0	-4.1	2.4	-14.1	-5.8

% Response Legend
x > 95%
95% ≤ x < 90%
90% ≤ x < 85%
85% ≤ x < 70%
x < 70%

Supplementary Dataset 2. Activity of PZ-2891 as a modulator of 72 proteins commonly associated with off-target drug interactions. The SAFETYscan was performed by DiscoverX using the indicated panel of GPCRs, ion channels, nuclear receptors, etc. at both 1 and 10 μM PZ-2891.
