# Supplementary material for: A therapeutic approach to pantothenate kinase associated neurodegeneration
Source: Nat Commun. 2018 Oct 23;9:4399. doi: 10.1038/s41467-018-06703-2 (PMC6199309; doi:10.1038/s41467-018-06703-2)
Supplement: Supplementary file 5 — Description of Additional Supplementary Files [file 41467_2018_6703_MOESM5_ESM.docx]

**Title:** Supplementary Dataset 1.

**Description:** Data supporting graphics presented in Supplementary Figure 7. The first Kinome screen yielded three hits (first DiscoverX primary screen report). These three hits were followed up in a second series of experiments and found to be false positives (second DiscoverX report).

**Title:** Supplementary Dataset 2.

**Description:** Activity of PZ-2891 as a modulator of 72 proteins commonly associated with off-target drug interactions. The SAFETYscan was performed by DiscoverX using the indicated panel of GPCRs, ion channels, nuclear receptors, etc. at both 1 and 10 μM PZ2891.
